# Cellular endosomal potassium ion flux regulates arenavirus uncoating during virus entry

**DOI:** 10.1128/mbio.01684-23

**Published:** 2024-06-14

**Authors:** Amelia B. Shaw, Hiu Nam Tse, Owen Byford, Grace Plahe, Alex Moon-Walker, Samantha E. Hover, Erica Ollmann Saphire, Sean P. J. Whelan, Jamel Mankouri, Juan Fontana, John N. Barr

**Affiliations:** 1 School of Molecular and Cellular Biology, Faculty of Biological Sciences, University of Leeds, Leeds, United Kingdom; 2 Astbury Centre for Structural Molecular Biology, University of Leeds, Leeds, United Kingdom; 3 Center for Infectious Disease and Vaccine Research, La Jolla Institute for Immunology, La Jolla, California, USA; 4 Department of Molecular Microbiology, Washington University in St. Louis, St. Louis, Missouri, USA; 5 Program in Virology, Harvard Medical School, Boston, Massachusetts, USA; Virginia Tech, Blacksburg, Virginia, USA

**Keywords:** arenavirus, LCMV, potassium, ion channels

## Abstract

Lymphocytic choriomeningitis virus (LCMV) is an enveloped and segmented negative-sense RNA virus classified within the *Arenaviridae* family of the *Bunyavirales* order. LCMV is associated with fatal disease in immunocompromised populations and, as the prototypical arenavirus member, acts as a model for the many highly pathogenic members of the *Arenaviridae* family, such as Junín, Lassa, and Lujo viruses, all of which are associated with devastating hemorrhagic fevers. To enter cells, the LCMV envelope fuses with late endosomal membranes, for which two established requirements are low pH and interaction between the LCMV glycoprotein (GP) spike and secondary receptor CD164. LCMV subsequently uncoats, where the RNA genome-associated nucleoprotein (NP) separates from the Z protein matrix layer, releasing the viral genome into the cytosol. To further examine LCMV endosome escape, we performed an siRNA screen which identified host cell potassium ion (K*^+^*) channels as important for LCMV infection, with pharmacological inhibition confirming K^+^ channel involvement during the LCMV entry phase completely abrogating productive infection. To better understand the K^+^-mediated block in infection, we tracked incoming virions along their entry pathway under physiological conditions, where uncoating was signified by separation of NP and Z proteins. In contrast, K^+^ channel blockade prevented uncoating, trapping virions within Rab7 and CD164-positive endosomes, identifying K^+^ as a third LCMV entry requirement. K^+^ did not increase GP-CD164 binding or alter GP-CD164-dependent fusion. Thus, we propose that K^+^ mediates uncoating by modulating NP-Z interactions within the virion interior. These results suggest K^+^ channels represent a potential anti-arenaviral target.

**IMPORTANCE:**

Arenaviruses can cause fatal human disease for which approved preventative or therapeutic options are not available. Here, using the prototypical LCMV, we identified K*^+^* channels as critical for arenavirus infection, playing a vital role during the entry phase of the infection cycle. We showed that blocking K*^+^* channel function resulted in entrapment of LCMV particles within late endosomal compartments, thus preventing productive replication. Our data suggest K*^+^* is required for LCMV uncoating and genome release by modulating interactions between the viral nucleoprotein and the matrix protein layer inside the virus particle.

## INTRODUCTION

The *Arenaviridae* family within the *Bunyavirales* order of enveloped segmented RNA viruses currently contains around 50 species grouped into four genera: *Antennavirus*, *Hartmanivirus*, *Mammarenavirus*, and *Reptarenavirus* (1, 2). Mammarenaviruses can be segregated into New World (NW) and Old World (OW) groups based on phylogeny, with both groups comprising several human-infecting arenaviruses responsible for fatal hemorrhagic fevers. Mammarenaviruses are associated with rodent hosts that act as stable reservoirs. Human disease typically results from spill-over events, in areas where human and rodent habitats coincide, but also occurs in nosocomial settings, providing a high risk to healthcare workers (3, 4). Important human pathogens from the NW group include Junín (JUNV), Machupo (MACV), and Guanarito viruses, alongside Lassa virus (LASV) and Lujo virus (LUJV), from the OW group. The OW lymphocytic choriomeningitis virus (LCMV) is associated with neurologic disease in immunocompromised individuals and is a well-known cause of miscarriages but is largely non-pathogenic to immunocompetent humans (5, 6). As such, LCMV represents a tractable model system for studying arenavirus molecular and cellular biology and acts as the prototype of the entire Arenaviridae family. Options for mitigation of arenavirus disease are limited, with very few effective preventative or therapeutic measures currently available.

The mammarenavirus genome comprises small (S) and large (L) RNA segments that use an ambisense strategy to encode a total of four proteins; the S segment encodes the nucleocapsid protein (NP) and the glycoprotein precursor (GPC), whereas the L segment encodes both the large protein (L), which is the catalytic component of the viral RNA-dependent RNA-polymerase, and the Z matrix protein (Z). GPC is co- and post-translationally cleaved by cellular proteases, yielding the stable signal peptide, GP1 and GP2, which exist together on the viral surface as tripartite trimers (7, 8). Arenavirus entry into cells is directed by GP1, responsible for receptor binding, and GP2, which promotes fusion between host and viral membranes (9). GP1 binds various cell surface receptors such as α-dystroglycan (LCMV and LASV) (10), neuropilin-2 (LUJV) (11), and transferrin receptor (JUNV and MACV) (12–14), although other alternate receptors have been implicated, including Tyro3/Axl/Mer and members of the T-cell immunoglobulin (TIM) and mucin receptor families (15, 16). Subsequently, arenaviruses are internalized through receptor-mediated endocytosis to traffic through the endocytic system. The changing biochemical composition of maturing endosomes induces structural changes to the GPC to trigger at least two critical events. The first is a receptor switch mechanism, in which GP1 binds an alternate endosomal-resident receptor, with LASV interacting with LAMP-1 (17, 18), LUJV with CD63 (11), and LCMV requiring CD164 (19, 20). The second is a conformational rearrangement of GP2, which exposes a fusion loop that mediates viral and cellular membrane fusion to release the genomic segments into the cytosol.

Endosomal acidic pH is a well-characterized trigger of enveloped virus entry, activating viral spikes, resulting in fusion between virion and endosomal membranes at characteristic locations within the endocytic system. However, it is emerging that other ions also act as entry triggers; for example, binding of calcium has been shown to promote entry of togaviruses (21), coronaviruses (22–24), and filoviruses (25), whereas potassium ions (K^+^) are required for uncoating of influenza virus by promoting separation of its matrix and ribonucleoprotein (RNP) components (26). Within the Bunyavirales order, we recently showed K^+^ expedited entry of Bunyamwera peribunyavirus (BUNV) and Hazara nairovirus (HAZV) by inducing structural changes in their virion spikes (27–29), promoting fusion between the virus and its target membranes (28, 30).

Here, we explored the ionic requirements for arenavirus multiplication. We performed an siRNA-mediated gene knockdown screen that showed arenavirus growth depended on several cellular K^+^ channels. Pharmacological inhibition corroborated these findings, with time-of-addition studies showing K^+^ channel activity was required during virus entry. K^+^ channel blockade trapped virions within Rab7-positive late endosomal compartments where virion uncoating was prevented despite the presence of second receptor CD164. Using a previously established CD164/GP1 binding assay, we showed the role of K^+^ in virion uncoating was not to promote this receptor interaction. We also performed a cell-cell fusion assay to show that K^+^ did not change the pH requirement for LCMV GPC-mediated fusion. Instead, our results are compatible with a role of K^+^ modulating interactions between virion components within the virion interior.

Taken together, these findings reveal an important role for K^+^ during LCMV entry and the opportunity for existing, licensed K^+^ ion channel inhibitors to be repurposed as a new, pharmacologically safe, and broad-ranging therapeutic strategy for arenavirus-mediated disease.

## RESULTS

### Generation of an eGFP-expressing LCMV variant

To investigate the dependence of LCMV on endosomal ion flux, an initial aim of this study was to perform a comprehensive siRNA screen of host cell ion channels involved in LCMV multiplication. To facilitate this, we generated a recombinant LCMV (rLCMV) expressing enhanced green fluorescent protein (eGFP) (rLCMV-eGFP) to permit live-cell monitoring of virus-specific gene expression.

First, to rescue wild-type rLCMV (rLCMV-WT), cDNAs pUC57-LCMV-S and pUC57-LCMV-L were designed to express wild-type LCMV-Armstrong (clone 13 derivative) S and L segment sequences, respectively (Fig. S1A). To confirm successful rescue, a silent *Xh*oI site was introduced into pUC57-LCMV-S to permit distinction of rescued rLCMV from non-recombinant LCMV stocks (Fig. S1B through E). To generate the rLCMV-eGFP variant, pUC57-LCMV-S was modified by insertion of a porcine teschovirus-1 2A peptide linker (P2A) sequence (31–33) between eGFP and LCMV NP open reading frames (ORFs; Fig. S2A). All viruses were rescued in BSR-T7 cells, with rescue confirmed by detection of NP by Western blot analysis of both transfected and infected cell lysates (Fig. S2B).

### rLCMV and rLCMV-eGFP exhibit similar growth kinetics

**Fig 1:**
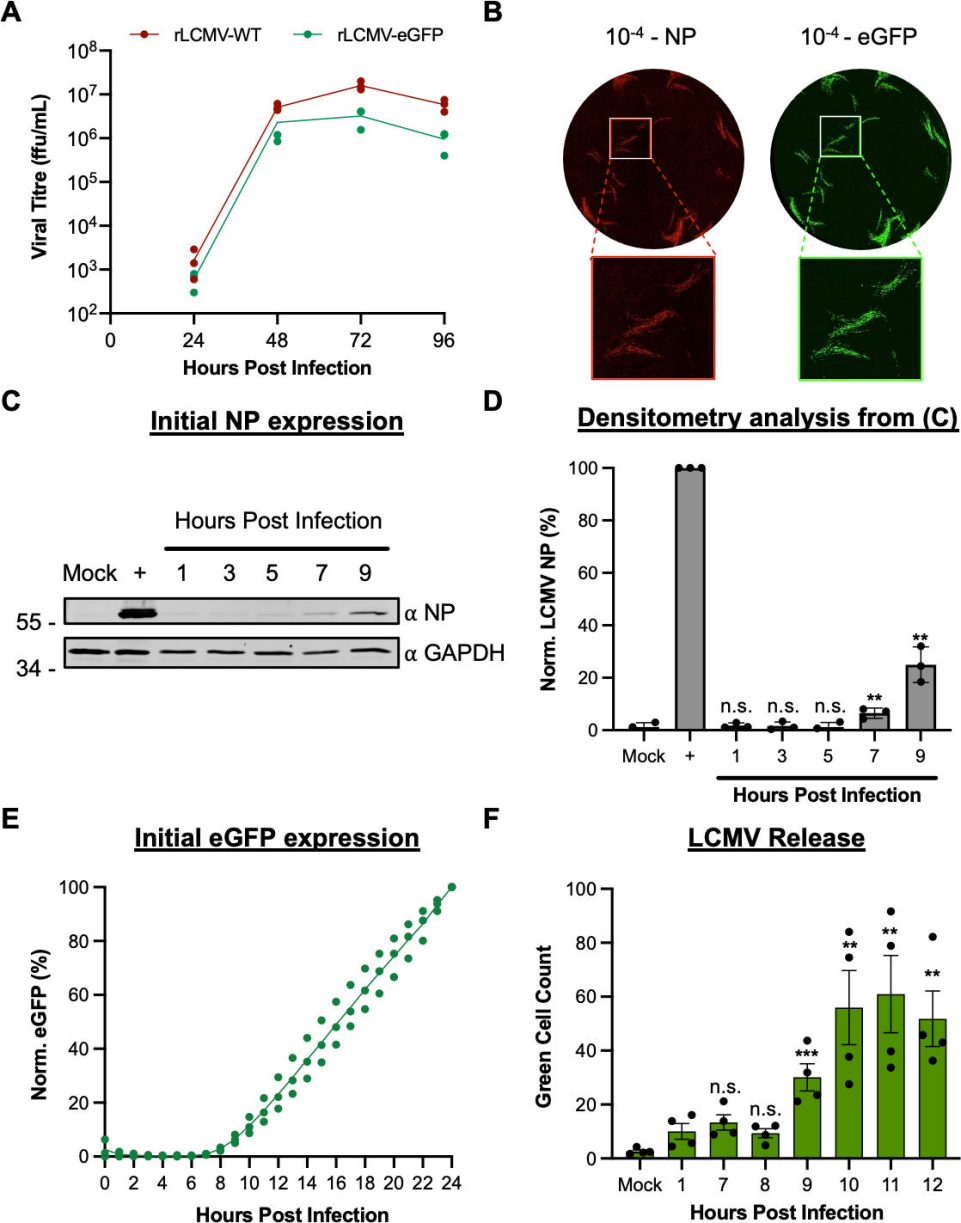
Comparison of replication kinetics of wild-type LCMV and eGFP-expressing LCMV. (**A**) BHK-21 cells were infected in triplicate (*n* = 3) with either rLCMV-WT or rLCMV-eGFP at an MOI of 0.001. Supernatant samples were collected every 24 h and were subsequently titered by focus-forming assay. The average titer of each time point was plotted with standard error. (**B**) Focus-forming assays for LCMV were performed by infecting BHK-21 cells with a serial dilution of the virus supernatant and incubating for 3 days. For rLCMV-WT, the focus-forming assay was fixed, permeabilized, and stained for LCMV NP by indirect immunostaining and was imaged using the IncuCyte S3 Live-Cell Analysis System. For rLCMV-eGFP, the focus-forming assays were imaged without fixing. (**C and D**) To identify initial synthesis of NP, SH-SY5Y cells were infected with rLCMV-WT, and lysates were collected at subsequent hours post-infection for Western blot (**C**) and densitometry analysis (**D**). This was plotted as a percentage of LCMV NP expression at 24 h post-infection, with standard error (*n* = 3). Results were analyzed by Student’s *t*-test, whereby n.s. denotes *P* > 0.5; *P < 0.05, **P < 0.01, ***P < 0.001, comparing the time points to NP expression at 24 h. (**E**) To examine time points of eGFP expression, SH-SY5Y cells were infected with rLCMV-eGFP, and the total integrated intensity of eGFP expression was measured every hour using the IncuCyte S3 Live-Cell Analysis System and plotted as a percentage of eGFP expression at 24 h, with standard error (*n* = 3). (**F**) SH-SY5Y cells were infected with rLCMV-eGFP at an MOI of 0.2 for 1 h at 37 ℃, after which the virus supernatant was removed, and the cells were extensively washed with phosphate-buffered saline (PBS). Fresh medium was added to the cells and incubated until the specified time point to transfer the supernatant to fresh cells, which were then imaged using the IncuCyte S3 Live-Cell Analysis System, 24 h after the time of transfer. The average number of eGFP-expressing cells counted (green cell count), resulting from four experimental repeats, is plotted with standard error. Results were analyzed by Student’s *t*-test, whereby n.s. denotes *P* > 0.05, **P < 0.01, ***P < 0.001, comparing time points to 1 h post-infection. n.s., not significant.

We next compared the multi-step growth kinetics of rLCMV-WT and rLCMV-eGFP to assess the fitness impact of the eGFP ORF and P2A linker. BHK-21 cells were infected with each virus at a multiplicity of infection (MOI) of 0.001, and released virus was measured at 24 h intervals by immunostaining focus-forming assay using NP antisera. This analysis revealed multiplications of rLCMV-WT and rLCMV-eGFP were similar, with less than 1-log difference between titers across the time course (Fig. 1A). Comparison between immunostained and fluorescent foci showed eGFP expression was an accurate surrogate marker for the detection of rLCMV-infected cells (Fig. 1B), with benefits of not requiring antibody staining and allowing tracking infection in live cells.

To better characterize NP and eGFP expression kinetics, human neuronal SH-SY5Y cells, relevant as neuronal cells are targeted during LCMV host infection, were infected with either rLCMV-WT or rLCMV-eGFP at an MOI of 0.1, and expression was measured at multiple hours post-infection (hpi). For rLCMV-WT, NP expression was first visible at 7 hpi (Fig. 1C and D). For rLCMV-eGFP, new eGFP expression was quantified through measuring the total integrated intensity of the eGFP (TIIE) signal, first detected at 8 hpi (Fig. 1E), suggesting the gene expression kinetics of these viruses were similar. Finally, to determine the time taken for a single replication cycle of rLCMV-eGFP, we performed a virus release assay. Cells were infected with rLCMV-eGFP, and at specified time points post-infection, all supernatants were transferred to fresh cells. The presence of infectious virus was then detected by incubating these fresh cells for 24 h, at which time the green cell count was taken to determine the number of eGFP-expressing cells. This analysis showed that the virus was first released from cells at 9 hpi (Fig. 1F), indicating this was the minimum time required for a complete rLCMV-eGFP multiplication cycle in these cells.

### Cellular K^+^ channels are required for LCMV infection

**Fig 2:**
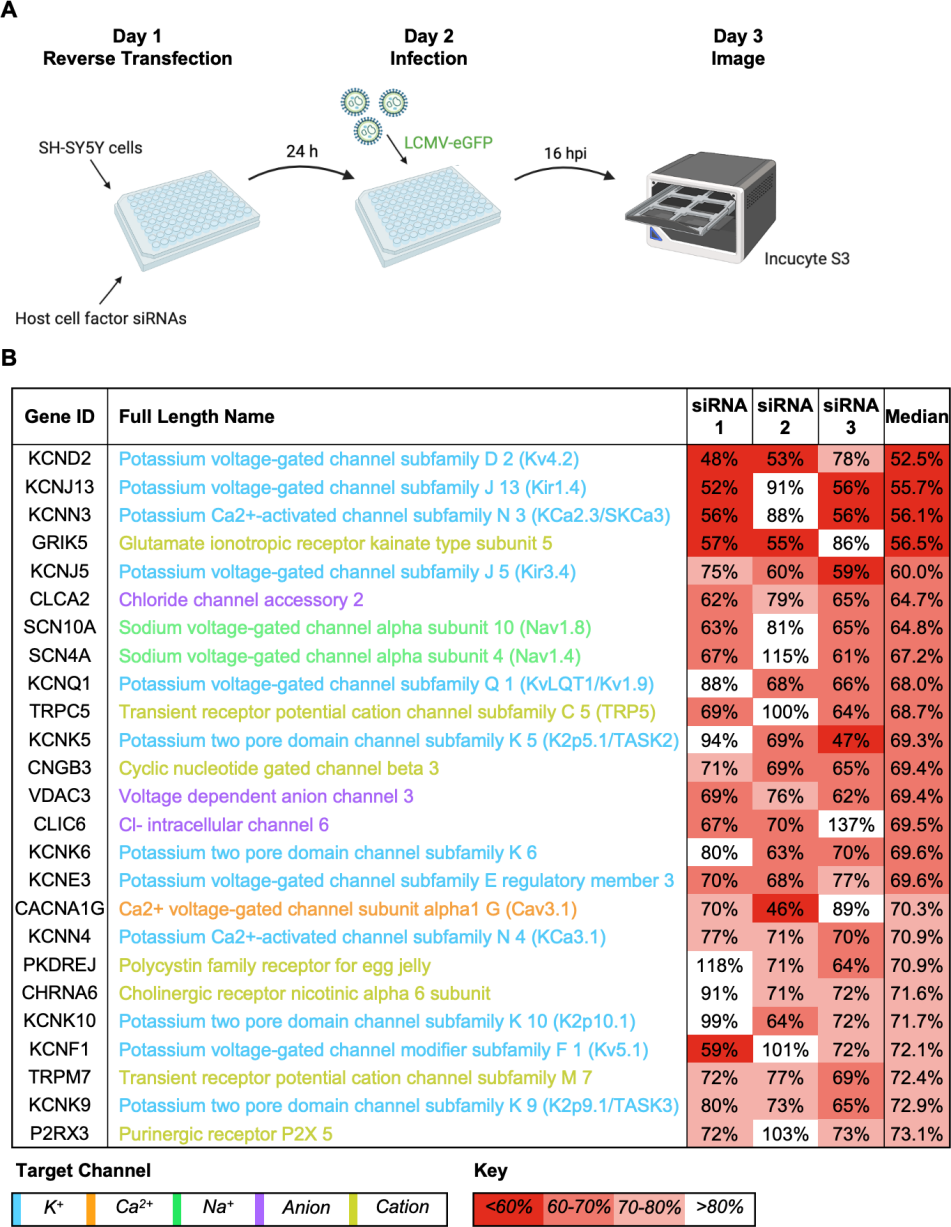
Assessment of the requirement of ion channels in the LCMV multiplication cycle using an siRNA screen. (**A**) Schematic depiction of the siRNA screening protocol. SH-SY5Y cells were reverse-transfected with siRNAs that targeted 176 cellular ion channel genes and then infected with rLCMV-eGFP at an MOI of 0.2. At 16 h post-infection (hpi), the cells were scanned using the IncuCyte S3 Live-Cell Analysis System for expression of eGFP. (**B**) The top 25 gene targets, which resulted in the highest knockdown of eGFP expression at 16 hpi, are shown. Percentage of eGFP expression is shown as the median of the average percentage of three individual siRNAs targeting the particular gene. Each percentage represents the mean of four experimental repeats. The channel families have been classified into color as shown by the target channel box. The percentage of expression of eGFP has been color coded with a red-to-white gradient, where red represents less than 60% eGFP expression and white represents more than 80% eGFP expression, as indicated by the key.

Next, we used rLCMV-eGFP to identify host cell ion channels that play a role during LCMV growth. To achieve this, we used a curated siRNA library comprising three unique RNA sequences targeting over 150 ion channel genes and assessed the ability of rLCMV-eGFP to multiply in the context of gene knockdown. Following reverse transfection of each unique siRNA, SH-SY5Y cells were infected with rLCMV-eGFP at an MOI of 0.2 for 16 h, after which the TIIE signal was quantified as a marker of successful rLCMV-eGFP infection (Fig. 2A). As previously determined, initial eGFP expression is first detected at 8 h, with rLCMV-eGFP first released from cells at 9 hpi (Fig. 1E and F). While virus will have been released from initially infected cells within the 16 hpi time point, eGFP expression in any newly infected cells will not be detectable. Thus, quantification of the eGFP signal recorded at 16 hpi would allow the maximum expression of eGFP while only reflecting the influence of siRNA knockdown on rLCMV-specific activities up to and including eGFP expression within the first round of infected cells. The influence of siRNA knockdown on later events in the growth cycle, including virus assembly, budding, and infection of new cells, would not be represented in the TIIE signal.

**Fig 3:**
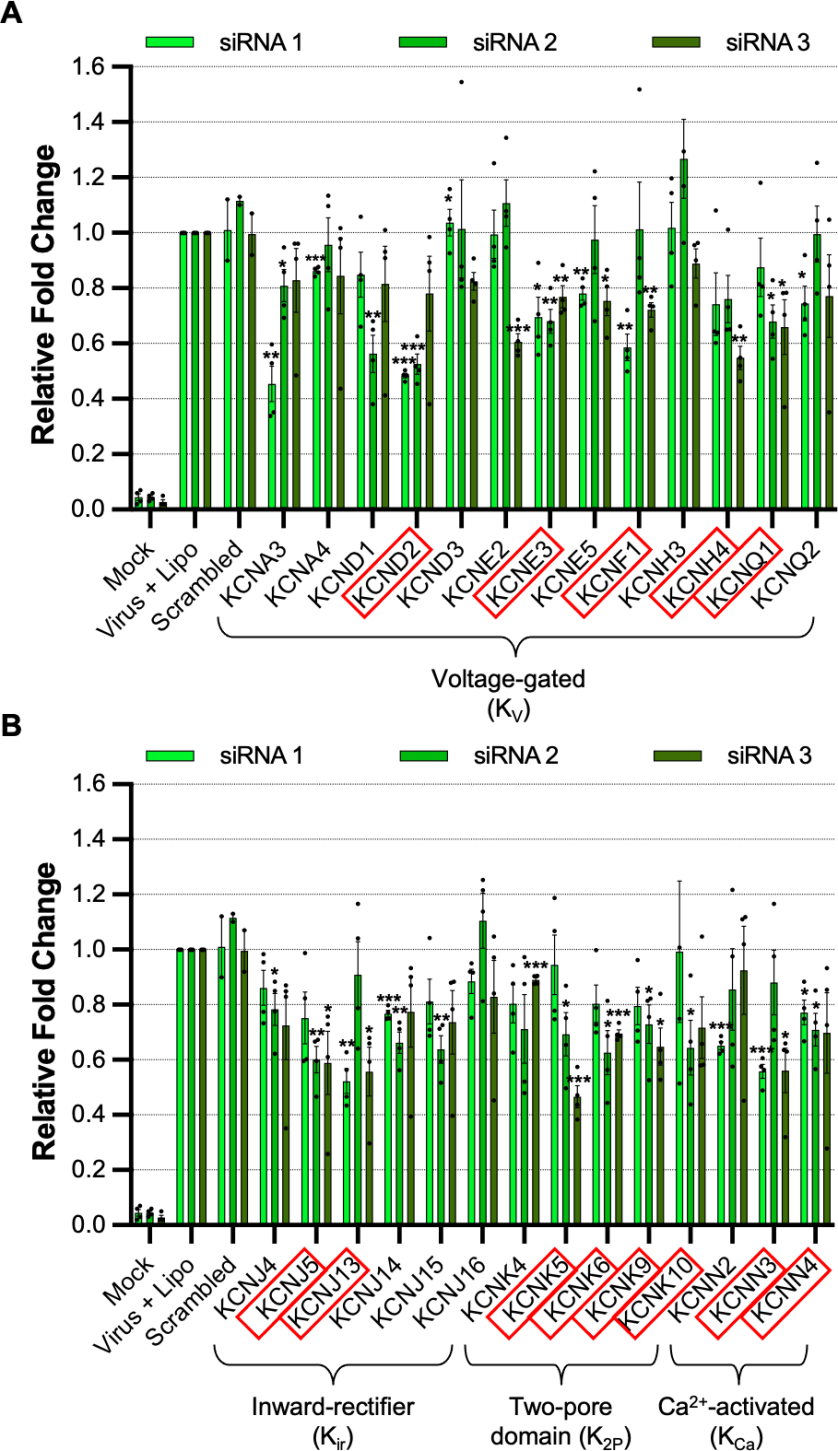
Cellular potassium ion channels are required for LCMV infection. Histograms showing the relative fold change of eGFP expression in SH-SY5Y cells after gene knockdown of a selection of potassium ion channels and infection with rLCMV-eGFP at an MOI of 0.2, after 16 h post-infection. The bars represent the knockdown eGFP expression of individual triplicate siRNAs, which targeted the same gene (siRNA 1, light green; siRNA 2, medium green; siRNA 3, dark green), as the result of four experimental repeats. Appropriate controls, such as mock-infected cells, cells infected with rLCMV-eGFP in the presence of a transfection reagent, and cells infected with rLCMV-eGFP after treatment with scrambled siRNAs, were included. Results were analyzed by Student’s *t*-test, whereby *P < 0.05, **P < 0.01, ***P < 0.001. (**A**) Histograms of siRNAs targeting genes expressing voltage-gated potassium (K_V_) ion channels. (**B**) Histograms of siRNAs targeting genes expressing inward rectifier (K_ir_), two-pore domain (K_2P_), and Ca^2+^-activated (K_Ca_) potassium ion channels. Red boxes were used to indicate the genes, the knockdown of which resulted in a reduction in eGFP expression below 75%.

The effect of each unique siRNA on TIIE was tested four times (Data Set S1), allowing the gene identities to be ranked according to their overall reduction in TIIE signal, calculated as the median of all three unique siRNA knockdowns against each gene target. The 25 cell channel genes with the greatest overall reduction in TIIE signal at 16 hpi are shown in Fig. 2B. Of these, 12 were K^+^ channels; 5 were voltage gated, 4 were from the two-pore family, 2 were calcium activated, and 2 were from the family of inward-rectifying K^+^ channels (Fig. 3). This information, taken together with the observation that four of the top five channels are involved in K^+^ conductance, suggests cellular K^+^ channels as a group are required for LCMV infection and highlights the importance of K^+^ in the LCMV multiplication cycle.

### Pharmacological blockade of K^+^ channels prevents LCMV infection

The findings of the siRNA screen showed knockdown of several cellular K^+^ channels resulted in significantly reduced rLCMV-eGFP-specific TIIE signal (Fig. 2 and 3). Given these data, we reasoned that blocking K^+^ channel function using pharmacological inhibitors would similarly reduce or prevent rLCMV-eGFP gene expression. To test this, we pretreated SH-SY5Y cells with a range of concentrations of broad-range K^+^ channel inhibitors, followed by infection with rLCMV-eGFP and measurement of TIIE signal at 24 hpi. Cell viability as an indication of compound toxicity was assessed by direct visualization of cell confluence and by 3-(4,5-dimethylthiazol-2-yl)-5-(3-carboxymethoxyphenyl)-2-(4-sulfophenyl)-2H-tetrazolium salt (MTS) assay. To eliminate the possibility that the selected inhibitors directly influenced eGFP processing or fluorescence, the effect of channel inhibition on rLCMV-WT NP gene expression was also measured in parallel by Western blot analysis, with glyceraldehyde 3-phosphate dehydrogenase (GAPDH) expression included as a loading control and to assess adverse effects of inhibitors on cell viability.

**Fig 4:**
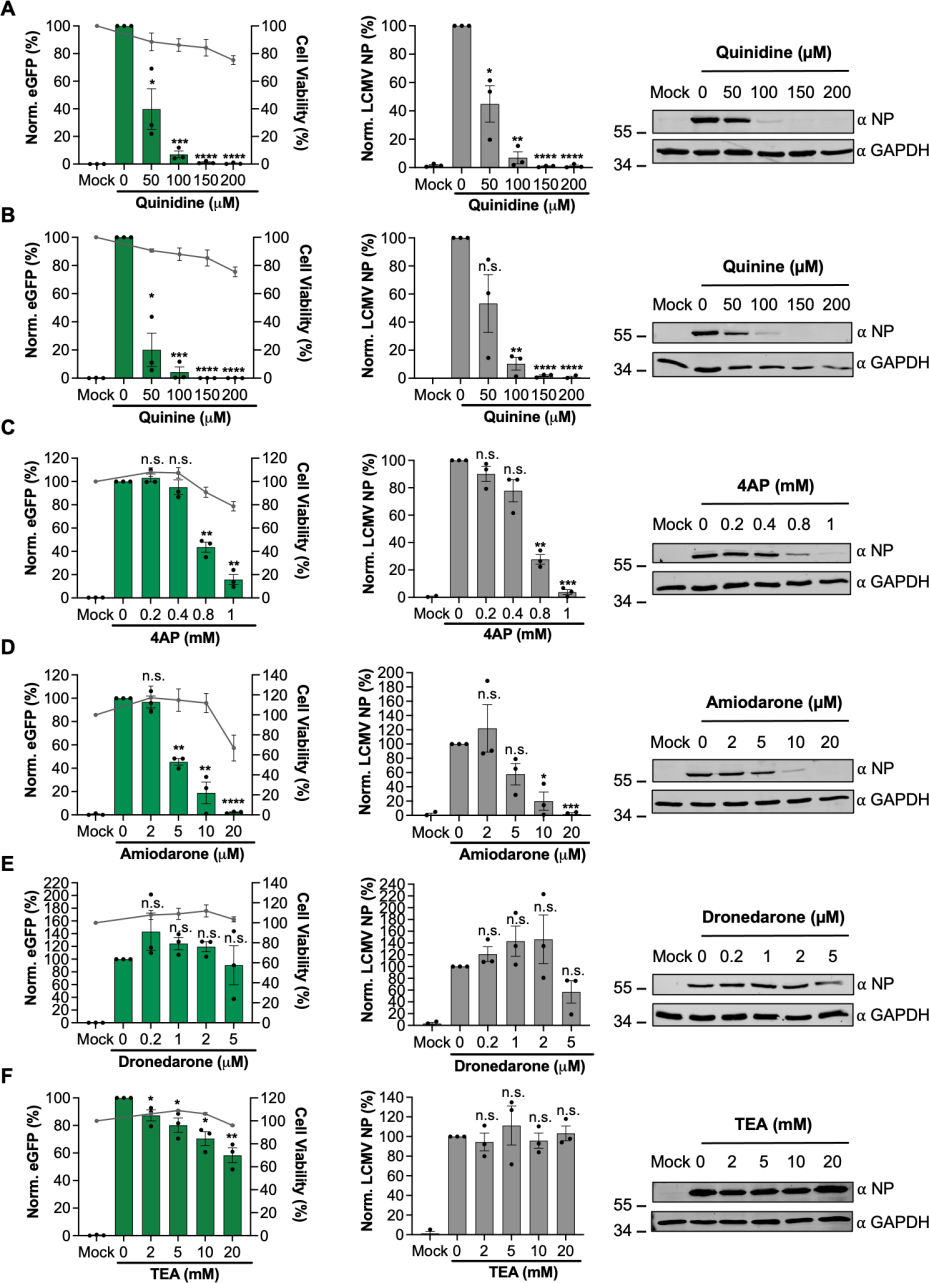
Broad-acting potassium ion channel inhibitors inhibit LCMV infection. (**A–F**) SH-SY5Y cells were pretreated for 45 min with increasing concentrations of broad-acting potassium ion channel inhibitors including TEA (**A**), quinidine (**B**), quinine (**C**), 4AP (**D**), amiodarone (**E**), and dronedarone (**F**). The cells were then infected in triplicate (*n* = 3) with either rLCMV-eGFP or rLCMV-WT at an MOI of 0.1 and were incubated for 24 h. eGFP expression (left-hand panels) was measured in live cells in triplicate (*n* = 3) using the IncuCyte S3 Live-Cell Analysis System and plotted as a percentage of cells infected with rLCMV-eGFP in the absence of the drug. Cell viability was also assessed using an MTS assay and plotted on the right axis. Cells infected with rLCMV-WT were lysed at 24 h post-infection, and the lysates were probed for NP expression. Densitometry analysis (middle panels) was performed on the Western blots in triplicate (*n* = 3) and plotted on the graphs as a percentage of cells infected with rLCMV-WT in the absence of the drug. Both eGFP expression and densitometry results were analyzed by Student’s *t*-test, whereby *P < 0.05, **P < 0.01, ***P < 0.001, comparing results to untreated controls. Examples of Western blots (right-hand panels) have also been included to demonstrate change in band size. 4AP, 4-aminopyridine; TEA, tetraethylammonium.

Quinidine, quinine, 4-aminopyridine (4-AP), and amiodarone all reduced rLCMV-eGFP signal and rLCMV-WT-specific gene expression levels in a dose-dependent manner at sub-toxic concentrations (Fig. 4, left and central panels). In particular, quinidine, quinine, and 4-AP each reduced rLCMV-WT-specific gene expression by over 90% compared to untreated cells, at sub-toxic inhibitor concentrations, revealing high potency (Fig. 4A through C, middle panels). Amiodarone (Fig. 4D), a Food and Drug Administration (FDA)-approved anti-arrhythmia drug with broad-range K^+^ channel blocking activity, also resulted in clear reduction in rLCMV-specific gene expression at non-toxic concentrations, reducing both rLCMV-specific TIIE and NP expression by approximately 80% that of untreated infected cells at non-toxic concentrations. Inhibition of rLCMV-specific gene expression by dronedarone (Fig. 4E) and tetraethylammonium (TEA, Fig. 4F) were less convincing, showing no significant reduction in either TIIE or NP expression. Taken together, these inhibitor studies support the importance of K^+^ channels in the LCMV multiplication cycle and further suggest that repurposing clinically approved drugs may be a viable approach to mitigate arenavirus growth and thus disease.

### The requirement of K^+^ channel activity acts during LCMV entry

**Fig 5:**
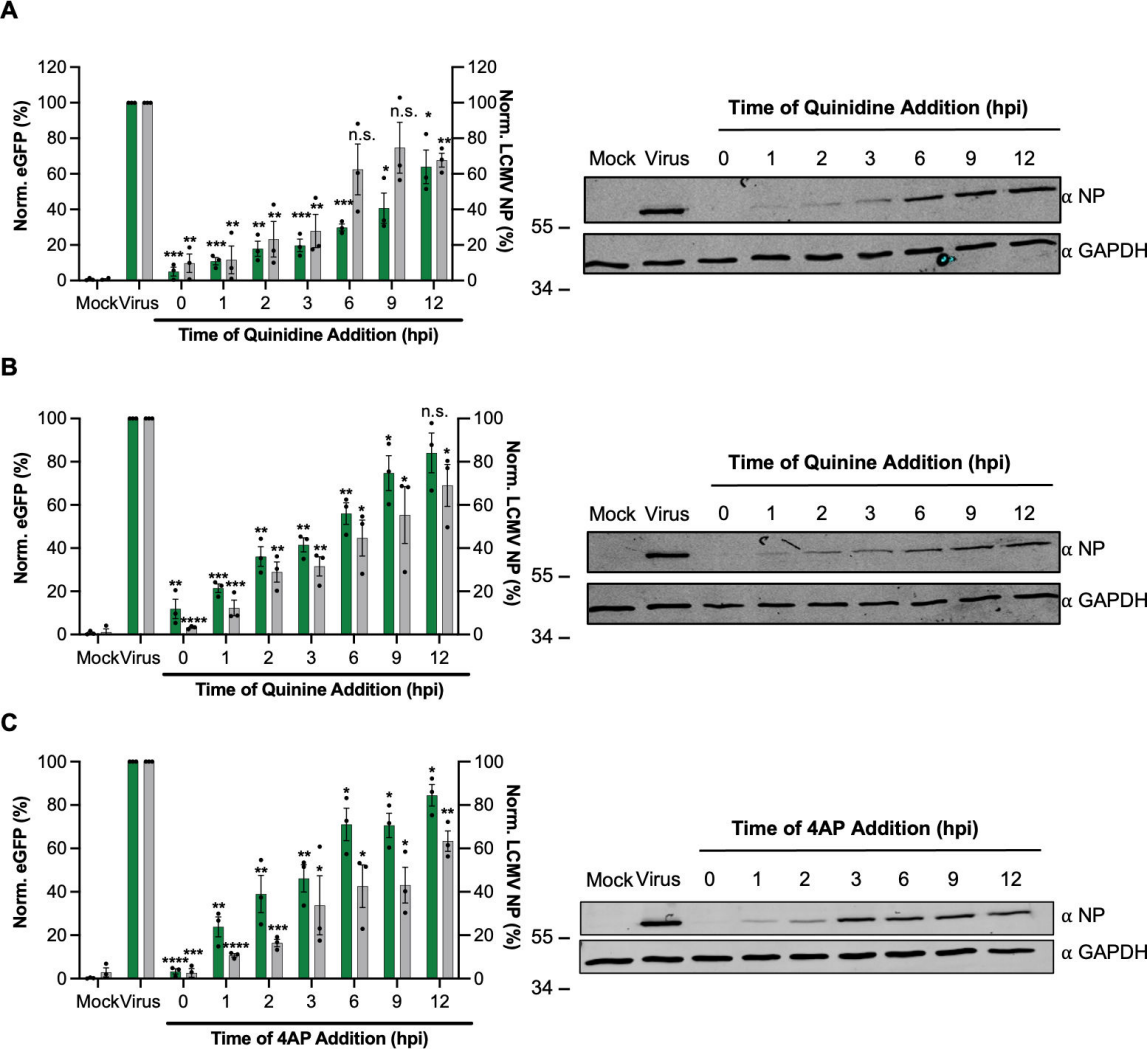
Cellular potassium ion channels are needed for early stages of LCMV infection. (**A–C**) SH-SY5Y cells were infected in triplicate (*n* = 3) with either rLCMV-eGFP or rLCMV-WT at an MOI of 0.1 and were incubated for 24 h. At the indicated hours post-infection (hpi; 0, 1, 2, 3, 6, 9, and 12 h), a suitable concentration of quinidine (A) (150 µM), quinine (B) (150 µM), or 4AP (C) (1 mM) was added for the remainder of the incubation time. At 24 hpi, the cells were then either imaged using the IncuCyte S3 Live-Cell Analysis System for analysis of eGFP expression or lysates were collected for Western blot and densitometry analysis. These were plotted as a percentage of untreated eGFP or NP expression at 24 h post-infection (virus, *n* = 3) (left-hand panels) and examples of Western blots are shown (right-hand panels). Results were analyzed by Student’s *t*-test, whereby n.s. denotes *P* > 0.05; *P < 0.05, **P < 0.01, ***P < 0.001, ****P < 0.0001, comparing results to the untreated control (virus). 4AP, 4-aminopyridine.

Having established important roles for K^+^ channels in rLCMV multiplication, we next wanted to better define the stage of the infection cycle where the activity of these channels was required. To do this, we performed time-of-addition studies using the broad-range K^+^ channel inhibitors quinidine, quinine, and 4-AP, shown above (Fig. 4A through C), to ablate rLCMV gene expression within defined temporal windows. Cells were infected with either rLCMV-eGFP or rLCMV-WT, and then treated with each of the three inhibitors at sub-toxic concentrations at spaced time points between 0 and 12 hpi, with gene expression of all infected cultures measured at 24 hpi using both Western blotting with NP antisera and live-cell eGFP detection (Fig. 5). The time points were chosen to encompass all stages of the multiplication cycle, from initial entry to assembly and egress.

In line with the pretreatment results described above (Fig. 4A through C), quantification of Western blots showed pretreatment of quinidine at 0 hpi blocked all rLCMV-WT NP gene expression (Fig. 5). Addition of quinidine at subsequent “early” 1, 2, and 3 hpi time points reduced rLCMV-WT NP expression to near undetectable levels, whereas addition of this inhibitor at the “late” time points of 6, 9, and 12 hpi had little impact. The findings were broadly similar for quinine and 4-AP, as well as corresponding TIIE measurements across all time points. Taken together, these findings suggest K^+^ channel activity is required at early stages of the LCMV replication cycle, most likely during virus internalization and passage through the endocytic system, where we previously showed pharmacological K^+^ channel blockade collapsed K^+^ gradients (27).

### K^+^ channel inhibition prevents LCMV endosome escape by blocking uncoating

To further examine why siRNA- and pharmacologically mediated K^+^ channel inhibition prevented LCMV multiplication, we wanted to determine the fate of LCMV virions when infecting under conditions of K^+^ channel blockade. We postulated that K^+^ channel blockade and the subsequent collapse of endosomal K^+^ gradients prevented virions reaching a cellular destination where efficient gene expression takes place. Our goal was to determine how this occurred. To do this, we modified pUC57-LCMV-L within our rLCMV rescue system to incorporate a HA-tag into the Z matrix protein open reading frame (Fig. S3A) (34). This strategy provided two distinct epitopes, Z-HA and NP, within each virus particle that we could detect with specific antisera, to allow both unambiguous assignment of co-localized IF signal to infecting virions and visualization of virus uncoating, when NP and Z proteins are proposed to spatially separate. The resulting plasmid (pUC57-LCMV-L-Z-HA) was used in subsequent rescue experiments, with generation of infectious virus bearing an HA-tagged Z protein, rLCMV-Z-HA, confirmed by detection of both NP and Z expression by Western blotting in both transfected BSR-T7 and infected BHK-21 cell lysates (Fig. S3B).

**Fig 6:**
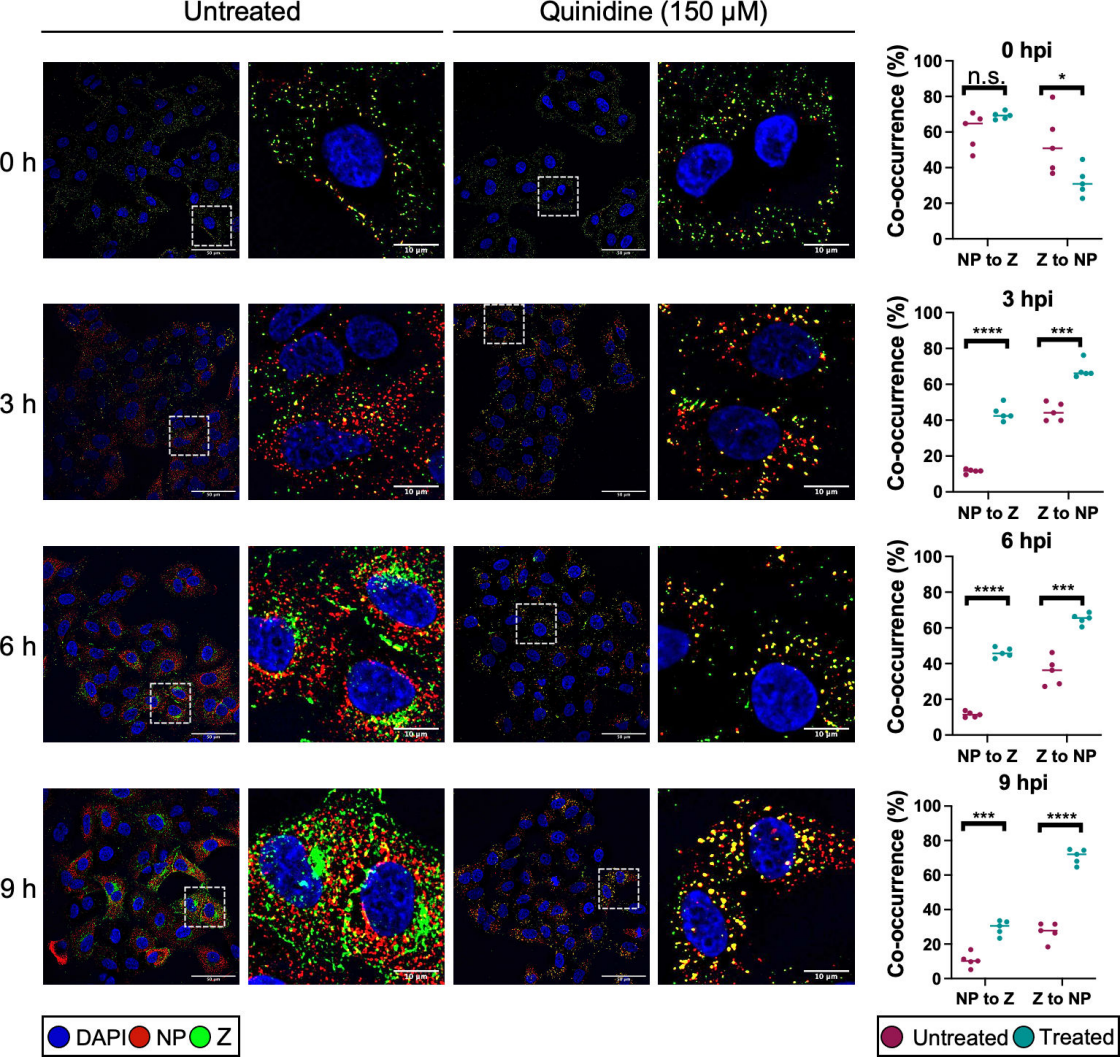
LCMV becomes trapped in presence of potassium ion channel inhibitors. A549 cells were untreated or pretreated with 150 µM quinidine and then infected with rLCMV-Z-HA at an MOI of 5 and fixed with formaldehyde at either 0, 3, 6, or 9 h. The cells were then permeabilized, blocked, and stained for the nucleus (DAPI, blue), LCMV NP (red), and Z-HA (green) by indirect immunostaining. The cells were then imaged on an Olympus IX83 widefield microscope at ×60 magnification. White dashed boxes were included to indicate which region of cells has been shown in the zoomed image. Scale bars representing 50 and 10 µm (zoomed images) are shown. Alongside the images is co-occurrence analysis, performed over five different images including >150 cells, which has been determined using the Manders coefficient method. The percentage of NP signal to Z signal and Z signal to NP signal was compared between untreated (pink) and quinidine-treated (blue). The co-occurrence analysis between untreated and treated was further analyzed through Student’s *t*-test, whereby n.s. denotes *P* > 0.05; *P < 0.05, ***P < 0.001; ****P < 0.0001. DAPI, 4',6-diamidino-2-phenylindole.

Human epithelial lung A549 cells were either untreated or pretreated with 150 µM quinidine before being infected with rLCMV-Z-HA at an MOI of 5 and fixed at subsequent time points (Fig. 6). Quinidine was chosen as a representative drug for examination by immunofluorescence microscopy. At 0 hpi, intact virions were identified on the cell surface shown by co-localization between NP signal in red and Z-HA signal in green, similar in both untreated and quinidine-treated cells. At 3 hpi, in untreated cells, the NP signal was separated from the Z-HA signal, consistent with virion disassembly and RNP release. At 6 hpi, signals for both NP and Z were increased in intensity, with amplification of Z signal delayed relative to NP, expected due to the ambisense mode of Z gene expression. Both NP and Z adopted a distinct cellular localization that became increasingly apparent at 9 hpi. In stark contrast, within quinidine-treated cells, the NP and Z-HA signals were predominantly found co-localized in punctate regions distributed throughout the cell at all time points examined. This punctate phenotype was also observed at 6 hpi for quinine and 4-AP (Fig. S4). This suggested that K^+^ channel inhibition and subsequent block of K^+^ influx had trapped incoming virions during the entry process, preventing uncoating. This further showed, for the first time, that arenavirus uncoating involves the separation of Z and NP during entry.

### LCMV virions are trapped in late endosomes after K^+^ channel inhibition

We next sought to identify the cellular location of LCMV virions which had become trapped upon K^+^ channel inhibition. To do this, cells were either untreated or pretreated with quinidine and infected with rLCMV-Z-HA at an MOI of 5 before fixing at 6 hpi. The 6hpi time point was chosen because it represented a clear difference in the distribution of NP and Z signals between untreated and treated cells, as shown in the previous section (Fig. 6). We co-stained the cells for NP and Z to identify intact virions, alongside cellular markers which represented different stages of the endocytic pathway: Rab5 for early endosomes, Rab7 for late endosomes, LAMP1 for lysosomes, and CD164 as an established LCMV late endosomal secondary receptor. Co-occurrence analysis was then performed on over 150 cells across five images. Then, to examine only points at which both the NP and Z signals were present, indicative of specific virions, the NP and Z signals were multiplied together (NP/Z), and the Manders overlap coefficient was calculated to determine what percentage of the NP/Z signal overlapped with the cell marker signal.

**Fig 7:**
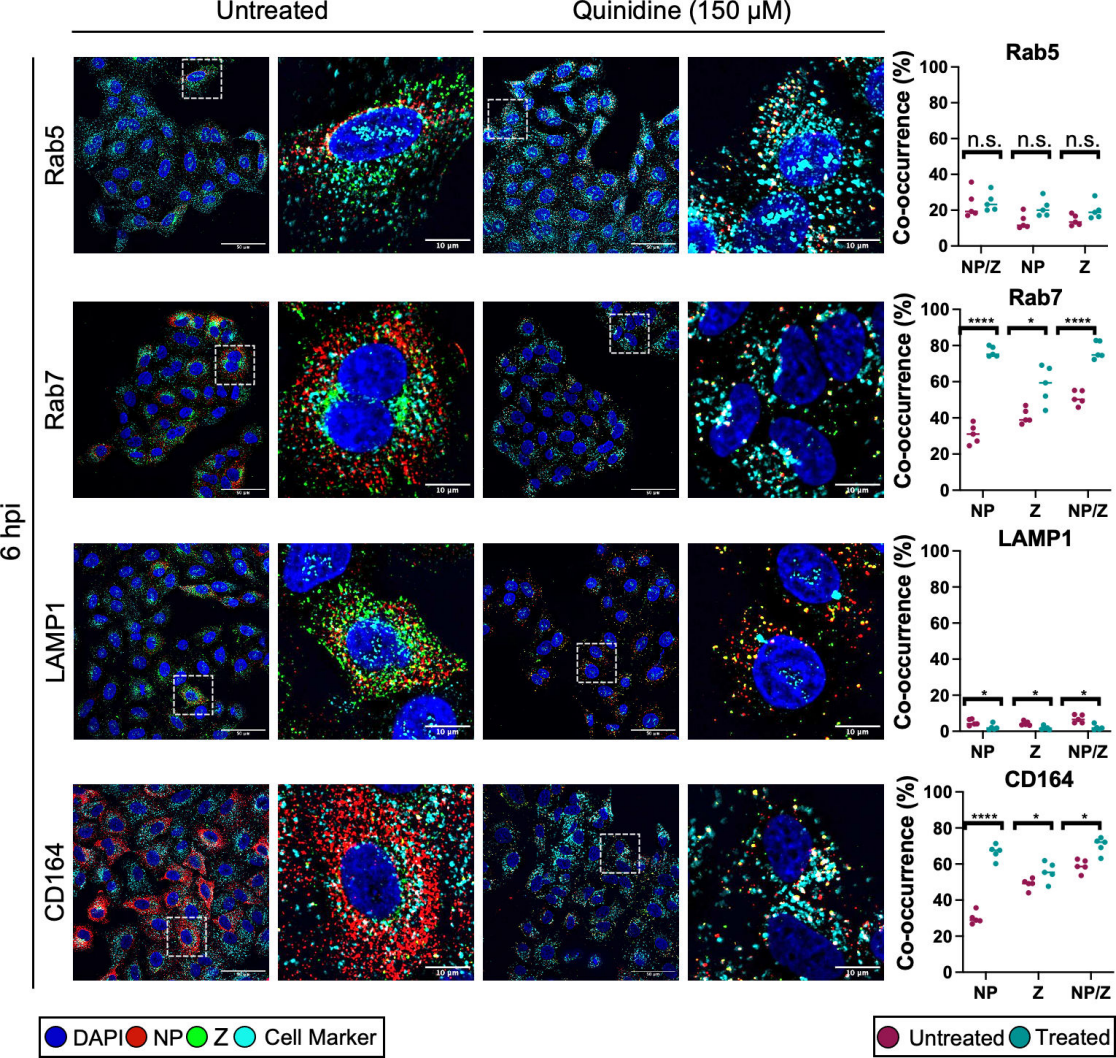
LCMV NP puncta co-localize with Rab7 and CD164 in cells treated with potassium ion channel inhibitors. A549 cells were untreated or pretreated with 150 µM quinidine and then infected with rLCMV-Z-HA at an MOI of 5 and fixed with formaldehyde at 6 h post-infection (hpi). The cells were then permeabilized, blocked, and stained for the nucleus (DAPI, blue), LCMV NP (red), Z-HA (green), and for cellular markers (cyan), Rab5, Rab7, CD164, and LAMP1, by indirect immunostaining. The cells were then imaged on an Olympus IX83 widefield microscope at ×60 magnification. White dashed boxes have been included to indicate which region of cells has been shown in the zoomed image. Scale bars representing 50 and 10 µm are included. Alongside the images is co-occurrence analysis, performed over five different images including >150 cells, which has been determined using the Manders coefficient method. The percentage of either NP signal, Z signal, or NP and Z signals has been examined against the signal of the marker. Comparison between untreated (pink) and quinidine treated (blue) was further analyzed through Student’s *t*-test, whereby n.s. denotes *P* > 0.05; *P < 0.05, ****P < 0.0001.

Our results showed that when cells were treated with quinidine to block endosomal K^+^ influx, there was a significant increase in the co-occurrence between NP/Z and Rab7 signals, resulting in approximately 70% of signal overlap (Fig. 7, second row of panels). A similar trend was also found when the co-occurrence analysis was performed on CD164 signal (~70% overlap between NP/Z and CD164 signals; Fig. 7, fourth row of panels). This suggested that there was a significant accumulation of trapped virions in Rab7-positive and CD164-positive late endosomes after quinidine treatment. A significant co-occurrence between the signals of NP/Z and Rab7, and NP/Z and CD164 was also observed when cells were treated with quinine and 4-AP (Fig. S4). Little co-occurrence was seen between NP/Z signal and that of Rab5 (~20%), and there was no significant increase in co-occurrence after quinidine treatment (Fig. 7, first row of panels). Furthermore, less than 5% of the NP/Z signal co-occurred with LAMP1 signal before or after quinidine treatment (Fig. 7, third row of panels). Taken together, these data suggest that when endosomal K^+^ influx is blocked through K^+^ channel inhibition, virions progress through Rab5-positive early endosomes, become trapped in Rab7/CD164-positive late endosomes, and do not reach LAMP1-positive lysosomes. Thus, LCMV RNPs cannot escape from the trapped virions, gene expression cannot occur, and infection is blocked.

### High K^+^ concentration does not affect the optimal pH for GP1-CD164 binding

The results of the previous section showed K^+^ channel blockade led to entrapment of LCMV in late endosomes, suggesting K^+^ is required for virion uncoating, and we were curious as to why this might be.

Late endosomal compartments supply the two known requirements for LCMV fusion and escape, namely, low pH (4.5) and abundant CD164. Despite this, under K^+^ channel blockade, LCMV becomes trapped and unable to uncoat. As the GPC-CD164 interaction at low pH is known to promote membrane fusion (19), we reasoned K^+^ may exert its influence on uncoating by one of three options: (i) by modulating the interaction between external virion component GP1 and its CD164 ligand; (ii) by modulating GPC-CD164-mediated membrane fusion; or (iii) by influencing separation between internal virion components such as RNP and Z (i.e., uncoating). This latter possibility is particularly intriguing, considering a similar mechanism proposed for influenza virus (26) and the recent demonstration that the arenavirus interior is accessible to ion influx via formation of a glycoprotein-associated envelope pore in response to low pH (35, 36).

**Fig 8:**
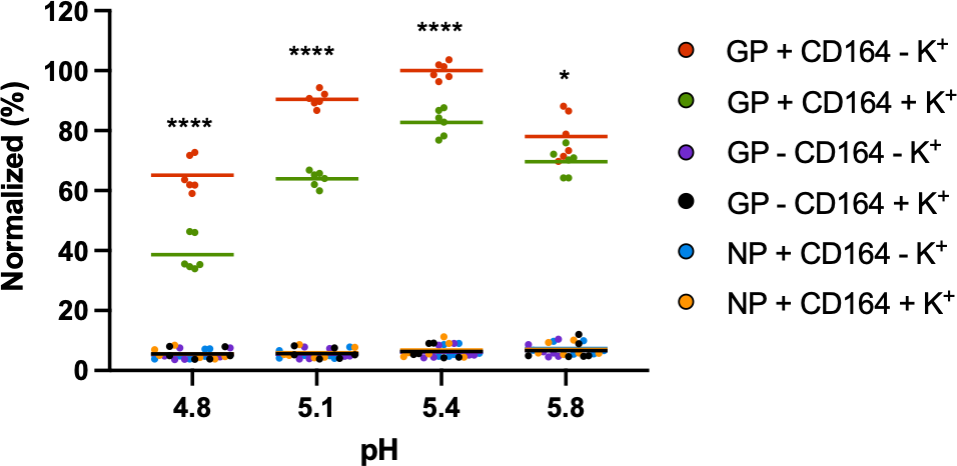
LCMV GP1 binding to CD164 is reduced in the presence of potassium. Soluble LCMV GP1 was used to coat 96-well high-protein-binding plates overnight, and then the wells were washed and blocked before washing in buffers at the specified pH, containing either 5 mM potassium chloride (GP+ CD164 – K^+^, red) or 140 mM potassium chloride (GP+ CD164+ K^+^, green). CD164-Fc was then added at the specified pH, washed, and incubated with goat anti-human IgG-HRP. The wells were washed and 3,3′,5,5′-tetramethylbenzidine (TMB) substrate was added, and the reaction was stopped with sulfuric acid, before reading the plate at 450 nm on a spectrophotometer plate-reader. The experiment was performed three times in duplicate with all results shown. All results were normalized to GP + CD164 – K^+^ at pH 5.4, which was the condition that resulted in the strongest absorption. Controls whereby LCMV NP was bound to the plate instead of GP1 (NP + CD164 – K^+^, blue, or NP + CD164 + K^+^, orange) or where no CD164 was added (GP – CD164 – K^+^, purple, or GP – CD164 – K^+^, black) were included to show lack of binding between NP and CD164 or between GP1 and goat anti-human IgG-HRP. The difference between 5 mM potassium chloride or 140 mM potassium chloride at the respective pH was further analyzed through Student’s *t*-test, whereby *P < 0.05, ****P < 0.0001.

To test the first option, whether K^+^ modulated the GP1-CD164 interaction, we utilized a previously described enzyme-linked immunosorbent assay (ELISA)-based binding assay (19), where immobilized LCMV GP1 was incubated with soluble CD164 at endo-lysosomal relevant pH values between 5.8 and 4.8, either in the presence or absence of K^+^. Interestingly, we found GP1-CD164 binding curves were similar, irrespective of K^+^ concentration, with maximal binding in both cases occurring at pH 5.4 (Fig. 8), consistent with the late-endosomal site of LCMV entry. This suggested the critical role of K^+^ in uncoating was not to promote the GP1-CD164 interaction.

### Exposure to high K^+^ ion concentration does not alter the pH threshold for LCMV GPC-mediated fusion

Due to the lack of influence of K^+^ on GP1-CD164 binding, we next sought to determine whether K^+^ was able to affect GPC-mediated membrane fusion, with previous work showing that a pH of 4.5 was optimal for this process (19).

**Fig 9:**
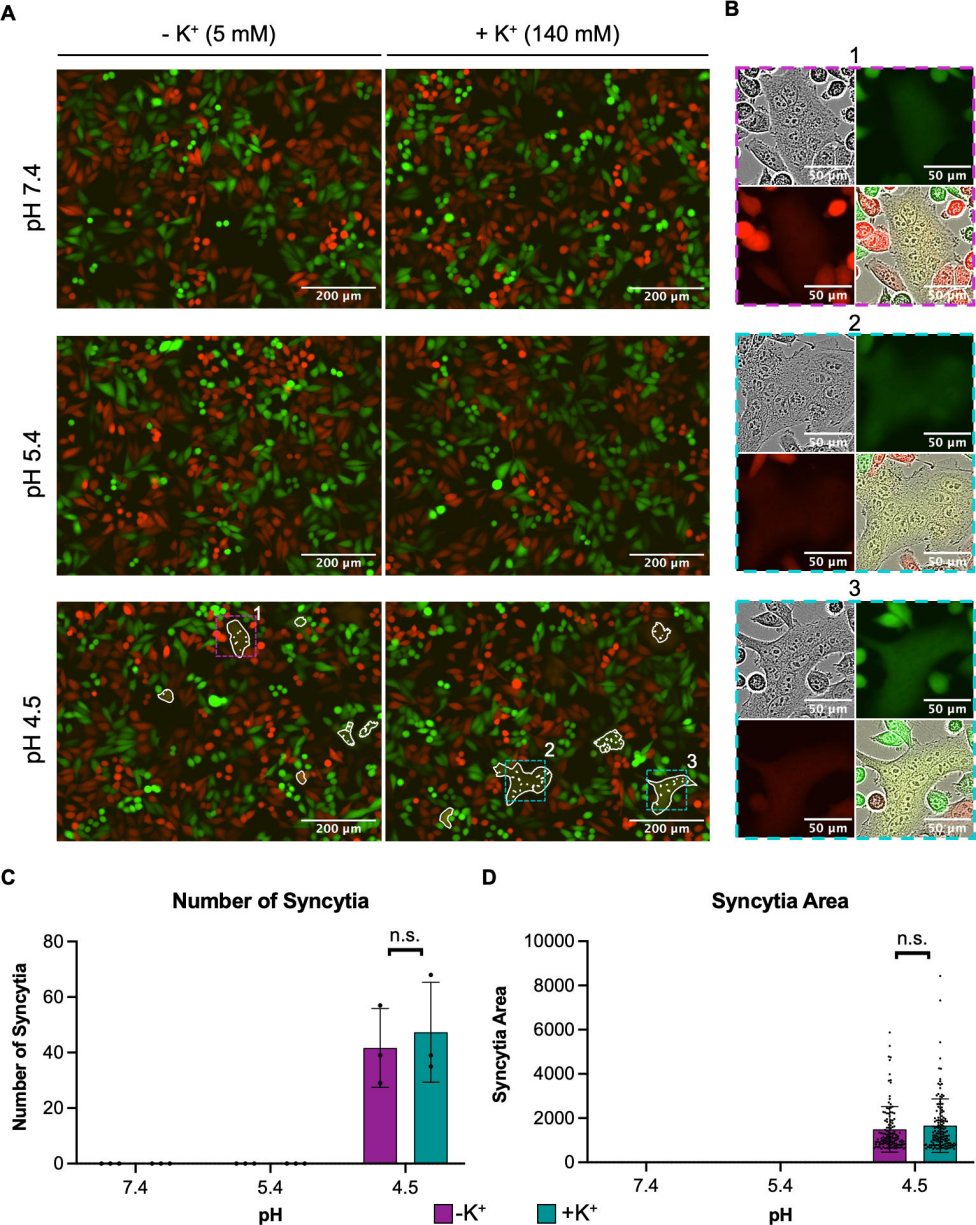
LCMV GPC and CD164-mediated fusion is not improved by the presence of potassium. (**A**) ∆CD164 HeLa cells expressing mCherry (red) were transfected with pCAGGS LCMV-GPC. At 4 h post-transfection, the cells were washed, trypsinized, and mixed 1:1 with ∆CD164 HeLa cells expressing plasma membrane-localized CD164 mutants and eGFP (green) to be seeded onto poly-L-lysine-coated 12-well plates. After overnight incubation, wells were initially imaged. Cells were then washed and treated with media at pH 7.4, 5.4, or 4.5 supplemented with 5 mM potassium chloride (−K^+^) or 140 mM potassium chloride (+K^+^) for 10 min. The treatment was then removed, and the cells were incubated in media supplemented with HEPES for 1 h. The cells were imaged using the IncuCyte S3 Live-Cell Analysis System. From three independent experimental repeats, representative images of green and red fluorescence are shown with a white outline indicating green and red fluorescence overlap and thus syncytia formation. (**B**) Zoom images of example syncytia indicated in panel **A **are shown as phase, green, red, and merge images. (**C**) Masks of green and red overlap areas were exported from the IncuCyte S3 program and imported into Fiji software. Here, the masks were made binary and inverted, and the number and areas of individual syncytia were measured. The number of syncytia seen across all images is plotted in panel** C**, whereas the average area of the syncytia is plotted in panel** D** alongside individual points representing the area of each syncytia. All three experimental repeats are shown. The difference between 5 mM potassium chloride or 140 mM potassium chloride at the respective pH was further analyzed through Student’s *t*-test, whereby n.s. denotes *P* > 0.05.

**Fig 10:**
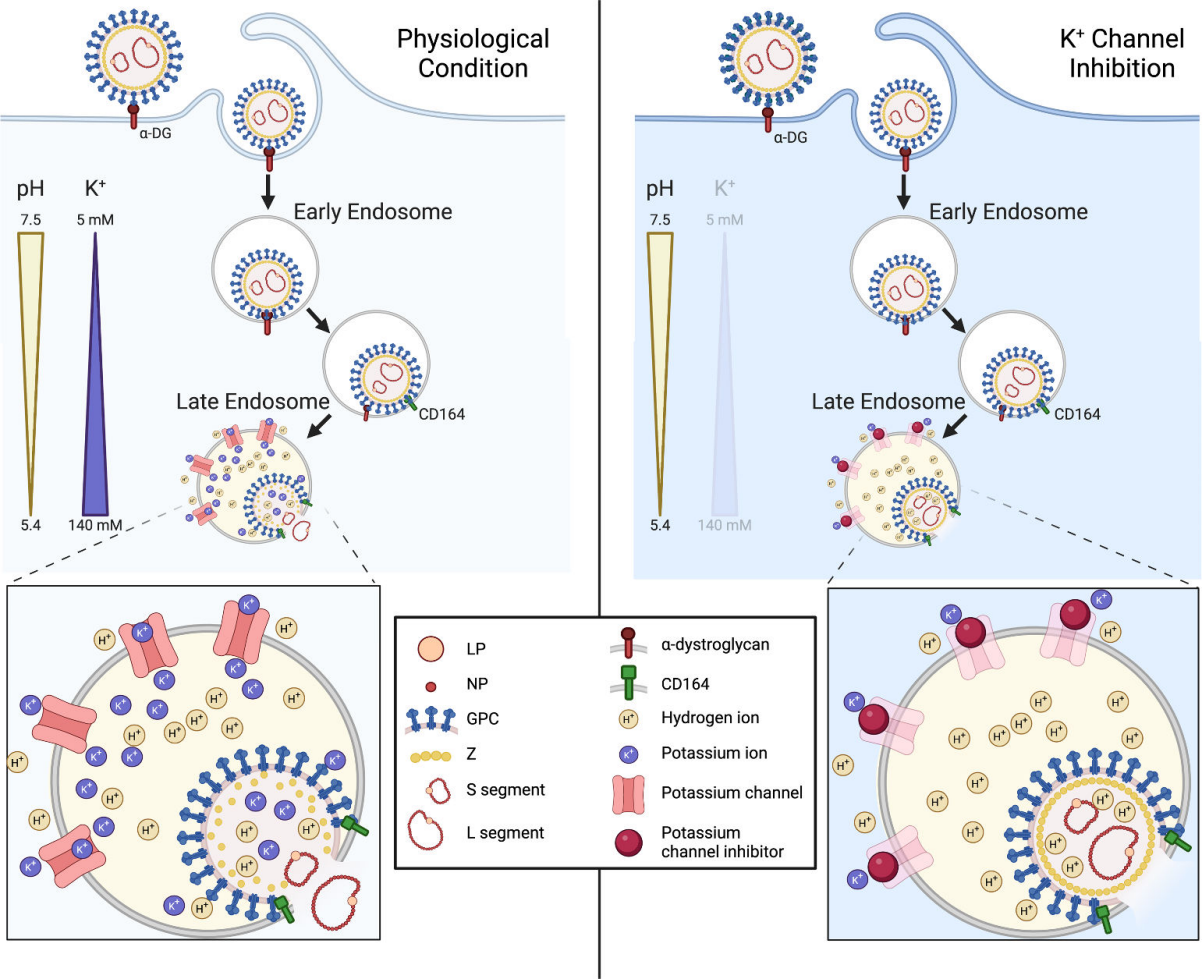
Proposed model of the role of potassium during LCMV entry. Proposed schematic model depicting the entry pathway of LCMV under physiological conditions and under conditions of potassium ion (K^+^) depletion. LCMV is internalized through macropinocytosis, after binding to alpha-dystroglycan, into early endosomes. LCMV then progresses through the endocytic system to late endosomes, where it experiences a reduction in pH and an increase in K^+^ concentration. The low pH is sufficient to drive a receptor switch to secondary receptor CD164, and the low pH and CD164 interaction drives membrane fusion and RNP release. We propose that, in addition to hydrogen ions (H^+^), K^+^ ions are transported into the virion interior to mediate destabilization and subsequent uncoating of the Z matrix layer. Under conditions of K^+^ depletion, we see incoming virions trapped in Rab7+ and CD164+ late endosomes, but we suggest that the absence of K^+^ in the endosome means no K^+^ is present in the virion interior, preventing uncoating of the viral matrix layer and release of the RNPs. This figure was created using Biorender. α-DG, α-dystroglycan.

We recreated the cell-cell fusion assay described previously (19) to examine the influence of K^+^ on fusion in the context of GPC bound to CD164. eGFP-expressing HeLa cells with surface-expressed CD164 were mixed with mCherry-expressing HeLa cells that expressed LCMV GPC, also on their surface. After exposing the cells to buffers with pH 7.4, 5.4, or 4.5 supplemented with low (5 mM) or high (140 mM) K^+^, we looked for evidence of syncytia formation, quantified by a mixing of red and green fluorescence, corresponding to fusion between CD164-expressing cells and LCMV GPC-expressing cells. In agreement with previous data (19), we observed an increase in the formation of syncytia when cells were exposed to pH 4.5, which was absent in cells treated with pH 7.4 (Fig. 9A, left panels). We also found that syncytia formation did not occur at pH 5.4 (Fig. 9A, left panels), supporting the previous proposal that fusion requires a low pH of 4.5. When we supplemented the pH buffers with K^+^, there was no syncytia formation at pH values of 7.4 and 5.4 (Fig. 9A, right panels), and there was no statistically significant change in the number of syncytia or the area of syncytia at pH 4.5 (Fig. 9C to D), indicating that K^+^ did not affect GPC fusion activity. Furthermore, these experiments were carried out on mock-transfected cells, and the lack of GPC meant there was no syncytia formation in any of the conditions (Fig. S5). Based on these results (Fig. 7 to 9) and those of others (26, 35, 36), we suggest K^+^ influences interactions between internal Z matrix and RNPs (Fig. 10).

## DISCUSSION

During viral entry to host cells, viruses encounter a complex environment with drastic changes in ionic concentration and membrane composition along the endocytic pathway (37). This includes a reduction in pH, concurrent with an increase in the concentrations of other ions. Previously, our group has demonstrated a role for K^+^ ions in the entry process of HAZV (28), in which K^+^ induces structural changes in the external viral glycoprotein spikes to form a fusion intermediate conformation. Additionally, for BUNV, we showed K^+^ has a direct effect on virion morphology, GP architecture, and function, beyond that elicited by acidic pH, with effects confined to the floor region of the GPC complex (30). The requirement of K^+^ for orthobunyavirus entry has further been demonstrated by others for La Crosse virus, Keystone virus, and Germiston virus (38, 39). To extend this work across the *Bunyavirales* order, we examined the role of K^+^ flux in the multiplication cycle of LCMV, the prototypic member of the *Arenaviridae* family. Using an eGFP-expressing LCMV variant generated through manipulation of an infectious clone, alongside both a siRNA screen targeting human ion channels and K^+^ ion channel inhibitors, we showed LCMV infection depends on K^+^ ion channel function.

Using recombinant LCMV bearing epitope-tagged components, we further pinpointed the role of K^+^ to the LCMV entry process, with K^+^ channel blockade resulting in entrapment of intact virions within late endosomes. Interestingly, not all broad-acting K^+^ channel inhibitors were effective at inhibiting LCMV infection, which may be the result of their different mechanisms of action. It is known that at physiological pH, quinidine, quinine, and 4-AP are all mostly protonated and are only able to cross the plasma membrane in their deprotonated form (40, 41). When these drugs are reprotonated in the cytosol, they can potently inhibit organellar K^+^ channels from the cytosolic side (40, 41). TEA, in contrast, is a quaternary ammonium cation and is membrane impermeable, and therefore will not be as effective in blocking organellar K^+^ channels (42, 43). This potentially explains why LCMV is unaffected by TEA because LCMV requires functional endosomal K^+^ channels and not K^+^ channels at the plasma membrane.

Here, we showed K^+^ channel blockade, thus preventing endosomal K^+^ influx, arrested LCMV in late endosomes where its two known entry requirements, namely, CD164 and fusion-enabling low pH of around 4.5, are encountered. This suggests K^+^ represents a third requirement for LCMV endosome escape, in addition to low pH and CD164. Thus, an important question becomes how does K^+^ act to promote endosome escape? In broad terms, LCMV endosome escape involves at least three distinct stages: interaction of GP1 and CD164, a GPC-mediated fusion step, and an uncoating step, where RNPs exit the confines of the virion matrix layer and are released into the cytosol, although whether these steps are sequential or concurrent is unknown. Bakkers et al. (19) used an ELISA to show that LCMV GP1 binds CD164 at low pH and a fusion assay to show that LCMV GPC binding CD164 at low pH is sufficient for the first envelope fusion step (19). Here, we used the same assays to show that K^+^ has no significant effect on either GP1-CD164 binding or GPC-mediated membrane fusion. Thus, by a process of elimination, our findings point toward a role for K^+^ in the virion uncoating step. Specifically, we propose this involves dissociation between RNPs and the Z matrix layer, in a similar way to that described for influenza virus (26). Our demonstration that LCMV NP and Z separate during entry, whereas they remain associated under K^+^ channel blockade, suggests that RNP release into the cytosol involves a K^+^-dependent dissociation from the Z matrix layer. To the best of our knowledge, this represents the first time this has been shown, which we depict in our model (Fig. 10).

K^+^-mediated virion uncoating is established for influenza virus, which involves K^+^ influx into the virion interior, facilitated by the M2 transmembrane viroporin (26). For influenza virus, K^+^ influx results in the dissociation of the matrix layer M1 that encases the eight RNP segments, which are then released into the cytosol for subsequent trafficking to the nucleus. In contrast, arenaviruses do not express a dedicated viroporin component as a functional analog of M2, and so the question of how K^+^ might penetrate the LCMV envelope to influence interior components is highly pertinent.

Interestingly, recent work suggests that the arenavirus envelope can be breached by H^+^ ions, resulting in acidification of the virion interior, measured using pH-sensitive indicator dyes in the context of lentiviral-based pseudovirus particles (35). Further to this, it has been proposed that arenavirus envelope permeability is mediated by structural rearrangements of the arenavirus glycoproteins themselves, following interactions with cellular factors at low pH (36). These structural rearrangements are proposed to lead to the formation of a transmembrane pore, allowing the passage of H^+^ into the virion interior as a critical entry trigger promoting virion uncoating. This model is similar to ours, with the additional requirement that K^+^ virion influx is needed for the uncoating step. Currently, the question of whether such a glycoprotein pore can allow the influx of K^+^ is untested, although as for influenza virus M2, it is reasonable to suggest that movement of ions might not be so selective. The functional parallels that necessarily exist between the entry of influenza virus and arenaviruses are interesting due to their shared possession of a matrix layer, which must be disassembled before RNPs can be released, and the fact that both viruses share viral fusion proteins of similar overall architecture (44). The results and model presented here suggest that the solution to this internal disassembly barrier has been solved in a similar way using viroporin-like activity.

This work supports a critical role for K^+^ ions in the LCMV infection cycle and represents the first study to establish the identity of cellular K^+^ channels that play a role in the arenavirus replication cycle. Interestingly, a previous report identified TRAM34 as a potent inhibitor of arenavirus entry, pointing toward a role for the TRAM34 therapeutic target, namely, the cellular calcium-activated K^+^ channel KCNN4 (KCa3.1) (45). However, the anti-arenaviral activity of TRAM34 was found to be independent of the ion channel target, and while the compound was found to act at the fusion stage of virus entry, the underlying mechanism of its activity remains unknown. It will be interesting to determine which of the several K^+^ channels that were identified in the LCMV siRNA screen are key contributors to endosomal K^+^ influx, with the aim of understanding how K^+^ influences both cellular and viral processes, as well as identifying host cell targets for anti-viral compounds. While our current investigations focus on K^+^ channel dependence in situations that favor acute infection outcomes, it would also be interesting to examine whether a common role of K^+^ channels also exists in situations that favor viral persistence, an outcome of LCMV infection of neonatal mice. Since cellular K^+^ channels are tested targets for clinically-approved human therapies, this raises the possibility that current licensed, FDA-approved K^+^ ion channel inhibitors could be repurposed for effective management of LCMV infection, or by extrapolation, to treat more serious arenavirus infections such as Lassa fever caused by LASV.

## MATERIALS AND METHODS

### Cell lines and virus

BHK-21 cells, BSR-T7 cells, SH-SY5Y cells, and A549 cells were acquired from American Type Culture Collection and maintained in high-glucose Dulbecco’s modified Eagle medium (DMEM, Sigma-Aldrich), supplemented with 10% heat-inactivated fetal bovine serum (FBS), 100 µg of streptomycin/mL, and 100 U of penicillin/mL, and incubated in a humidified incubator at 37°C with 5% CO_2_. ΔCD164 + mCherry HeLa cells and ΔCD164 + hCD164^PM^ + eGFP HeLa cells have been previously described (19) and were maintained under the same conditions as the aforementioned cell lines. BSR-T7 cells constitutively expressing T7 RNA polymerase (T7P) (46) were also additionally supplemented with G418 (500 µg/mL) every other passage to maintain the T7P plasmid. LCMV strain Armstrong virus and sequences were obtained from the National Collection of Pathogenic Viruses (1805291v), managed by the UK Health Security Agency.

### Plasmids

Full-length cDNAs representing the intact S and L segments were designed as previously reported (47, 48) and synthesized (GENEWIZ) using the LCMV strain Armstrong: clone 13 derivative (GenBank accession numbers: DQ361065 and DQ361066 for S and L, respectively) in pUC57, subsequently named pUC57-S and pUC57-L.

Additional supporting plasmids pUC57-NP and pUC57-LP were constructed using PCR amplification to sub-clone the NP ORF and the LP ORF from pUC57-S and pUC57-L, respectively, into the pUC57 vector using complementary flanking restriction sites.

Plasmid pUC57-S-eGFP was synthesized by sub-cloning the eGFP-P2A ORF (GenBank accession number: QFU20120.1, GENEWIZ) into pUC57-S using complementary flanking restriction sites. The eGFP-P2A ORF was inserted between the start codon of the NP ORF and the 3′ end of the untranslated region of the S segment. The eGFP ORF was separated from the NP ORF by the porcine teschovirus 1 2A self-cleaving peptide sequence (ATNFSLLKQAGDVEENPGP) (GenBank accession number: MH358390). Plasmid pUC57-L-Z-HA was synthesized through PCR insertion of the HA tag (YPYDVPDYA) at the end of the Z ORF, prior to the terminal stop codon. All primer sequences are available upon request.

### Generation of recombinant LCMV from cDNA

BSR-T7 cells were seeded in six-well plates at a density of 2 × 10^5^ cells/well. Following overnight incubation, the cells were transfected with 1.6 µg of pUC57-S or pUC57-S-eGFP, 1.6 µg of pUC57-NP, 2.8 µg pUC57-L or pUC57-L-Z-HA, 2 µg of pUC57-LP, and 0.6 µg of pUC57-T7 in 200 µL OptiMEM media, followed by 2.5 µL/µg TransIT-LT1 transfection reagent (Mirus). Control transfections were also performed, in which pUC57-L and pUC57-LP were excluded. At 24 hpt, the media were removed and replaced with 1 mL of DMEM supplemented with 2% FBS. At 120 hpt, the supernatants were collected, clarified, and used to infect BHK-21 cells, seeded the previous day at 2 × 10^5^ cells/well in six-well plates.

### Focus-forming assay

Focus-forming assays were performed on confluent monolayers of BHK-21 cells. Serial dilutions of LCMV (10-fold) made in serum-free DMEM were added to the cells and incubated for 1 h at 37°C. The dilutions were removed from the cells and overlaid with 0.8% methylcellulose. After 3 days’ incubation at 37°C, the overlay was removed, and the cells were fixed with 4% formaldehyde in phosphate-buffered saline (PBS), permeabilized with 0.3% Triton-X-100 and blocked using 1% bovine serum albumin (BSA). The cells were then incubated for 1 h with anti-LCMV NP polyclonal antibody (1:1,000), made in 1% BSA. The cells were then stained for 1 h at room temperature with an anti-sheep Alexa Fluor secondary antibody (Cell Signaling Technology). The entire well was then imaged at ×4 magnification using an Incucyte S3 automated microscope (Sartorius). Foci were counted and the titer was determined.

### Western blot analysis

Cells were lysed with 1× radioimmunoprecipitation assay buffer (50 mM Tris-HCl, pH 7.5, 150 mM NaCl, 1% (vol/vol) NP40 alternative, 0.5% (wt/vol) sodium deoxycholate, and 0.1% (wt/vol) sodium dodecyl sulfate (SDS), supplemented with 1× cOmplete, Mini, EDTA-free Protease I inhibitor cocktail (Sigma-Aldrich) for 15 min on ice. Lysates were collected; proteins were resolved on a 12% SDS-PAGE gel and then transferred to a polyvinylidene fluoride membrane. The transfer was performed at 15 V for 30 min using the Trans-Blot turbo (Bio-Rad). After transfer, the membrane was blocked for 1 h in Odyssey blocking buffer (PBS) (Licor, diluted 1:1 with 1× PBS). Subsequently, the membrane was stained with the primary antibodies (made in 1:4 blocking buffer:PBS supplemented with 0.1% Tween 20 detergent [PBS-T]; anti-LCMV NP 1:1,000; anti-GAPDH 1:7,500; anti-GFP [B-2] 1:1,000; and anti-HA Tag 1:1,000) for 1 h rocking, at room temperature and then with corresponding secondary antibodies (made in 1:4 blocking buffer:PBS-T; all secondary antibodies used at 1:10,000) for 1 h at room temperature. The membrane was dried and visualized on the Licor Odyssey Sa Infrared imaging system. Densitometry analysis was performed using ImageJ over three independent experiments.

### Assessment of viral release

SH-SY5Y cells (5 × 10^4^ cells/well) were seeded into a 48-well plate and incubated overnight. The cells were then washed with PBS and infected with rLCMV-eGFP at an MOI of 0.2 for 1 h at 37°C. After the incubation period, the infecting media were removed and washed four times with PBS before adding DMEM (supplemented with 2.5% FBS). At given time points, the supernatant from infected cells was removed and added to fresh cells (transfer cells). This was also performed for cells that were mock infected. The original infected cells were imaged for eGFP expression with the IncuCyte S3 Live-Cell Analysis System to confirm all wells had been infected with an equal amount of virus. The transfer wells were imaged for eGFP expression with the IncuCyte S3 Live-Cell Analysis System every hour for 24 h following the last time point. Analysis was performed on the images taken at 24 h after the supernatant from the infected cells was added to the transfer cells.

### siRNA screen

A master mix of 0.3 µL of Lipofectamine RNAiMAX reagent (Invitrogen) and 16.7 µL of Opti-MEM per well was pipetted into each well of a 96-well plate. Each siRNA from the 33 Silencer Human Ion Channel siRNA Library (Invitrogen) was transferred into the Opti-MEM mixture at a final concentration of 3 pmol. This was incubated for 20 min, after which 100 µL of a cell suspension of 2 × 10^5^ SH-SY5Y cells/mL in 10% DMEM was added to each well. Cells were incubated with the transfection and siRNA mix for 24 h at 37°C, following which 60 µL of the medium was removed and replaced with 200 µL of fresh 10% FBS DMEM to dilute the siRNAs and the transfection reagent. After a further 6 h, the medium was removed, and cells were washed in PBS, infected with rLCMV-eGFP at an MOI of 0.2, and incubated at 37°C until 16 hpi, when the eGFP expression was analyzed using the IncuCyte S3 Live-Cell Analysis System. The TIIE (green count units × μm^2^/image) was first normalized to confluency per well and then analyzed as a percentage of the total green integrated intensity in positive control wells containing the virus and lipofectamine only. Normalized values were averaged between two technical repeats and two biological repeats.

### Infection drug assays

SH-SY5Y (1 × 10^5^) cells/well were seeded into 12-well plates and incubated overnight. Cells were preincubated for 45 min with media supplemented with the specific ion channel inhibitor or the solvent control of each inhibitor (quinidine was prepared as a 100 mM stock in dimethyl sulfoxide [DMSO]; quinine was prepared as a 100 mM stock in water; 4-aminopyridine was prepared as a 100 mM stock in water; amiodarone was prepared as a 10 mM stock in DMSO; dronedarone was prepared as a 25 mM stock in DMSO; and tetraethylammonium was prepared as a 1 M stock in water). Cells were then incubated for 1 h with rLCMV-eGFP or rLCMV-WT at an MOI of 0.1, which was then replaced with the media from the preincubation. The cells were then incubated for a further 24 h before analysis of eGFP expression or NP expression by Western blot analysis.

### Time course of drug addition

LCMV-infected SH-SY5Y cells were treated with quinidine (150 µM), quinine (150 µM), or 4AP (1 mM) during the 24 h infection period (*T* = 0), or 1, 2, 3, 6, 9, and 12 hpi were added. Infection was allowed to proceed for a total of 24 h, and cells were analyzed for eGFP expression or NP expression by Western blot analysis.

### Immunofluorescence analysis: widefield

To assess localization of LCMV proteins during entry, rLCMV-Z-HA-infected A549 cells were grown on glass coverslips. At specified time points, the cells were fixed with 4% formaldehyde for 10 min, permeabilized with 0.3% Triton-x-100 for 10 min, and blocked with 1% BSA for 1 h. Cells were then labeled with anti-LCMV NP (1:500), anti-HA (for Z staining; 1:500), and antibodies against cell markers (Rab5, 1:200; Rab7, 1:100; LAMP1, 1:100; and CD164, 1:200) for 1 h, followed by labeling with corresponding Alexa Fluor 594, 488 or 647 nm secondary antibodies (all secondary antibodies used at 1:500), respectively, for 1 h. Cells were then washed and mounted onto microscope slides using ProLong gold containing DAPI (Invitrogen-Molecular Probes). Stained cells were viewed on the Olympus IX83 widefield microscope at ×60 magnification. Z stack images were collected, and the slice showing the strongest signal was chosen for the display image. Five randomly chosen fields were imaged for each condition, with a minimum of 25 cells in each field, to provide statistical co-occurrence analysis of >150 cells. Co-occurrence analysis was performed using an Otsu threshold generated in ImageJ software, and the Manders coefficient method was performed to determine co-occurrence.

### Enzyme-linked immunosorbent assay

The ELISA used here was adapted from Bakkers et al (19). High protein-binding MaxiSorp plates (ThermoFisher Scientific) were coated with 200 ng/well soluble LCMV sGP resuspended in 1× Tris-buffered saline (TBS) and incubated overnight at room temperature. Wells were then washed with 1× TBS and incubated with 3% BSA at 37°C for 1 h. Wells were extensively washed in ELISA buffer composed of 0.1 M citrate buffer, 3% BSA, 0.05% Tween 20, 1 mM calcium chloride, 1 mM magnesium chloride, 150 mM sodium chloride, and either 5 or 140 mM potassium chloride at the respective pH values. Soluble Fc-tagged CD164 (200 ng/well) in respective ELISA buffers was added and incubated at 37°C for 1 h. Wells were then extensively washed in ELISA buffer and incubated with goat anti-human IgG conjugated to horseradish peroxidase (1:500) in the respective ELISA buffers for 1 h at 37°C. Wells were once again washed extensively with ELISA buffer at the respective pH. 3,3′,5,5′-Tetramethylbenzidine ELISA substrate (ThermoFisher Scientific) was added for 15 min, followed by equal volumes of 2 M sulfuric acid to stop the reaction. The optical density at 450 nm of each well was read using a spectrophotometer plate reader.

### Cell-cell fusion assay

The cell-cell fusion assay mimicking LCMV fusion has been previously described (19). Briefly, ΔCD164 + mCherry HeLa cells had CD164 expression knocked out and were lentivirally transduced to express mCherry. ΔCD164 + hCD164^PM^ + eGFP HeLa cells had CD164 expression knocked out and were lentivirally transduced to express eGFP and a variant CD164, whereby the NYxxL lysosomal targeting motif was removed to increase cell plasma membrane expression. ΔCD164 + mCherry HeLa cells were seeded into a flask and then mock-transfected or transfected with a plasmid encoding LCMV-GPC using TransIT-LT1 transfection reagent (Mirus) following the manufacturer’s protocol. At 4 h post-transfection, cells were trypsinized, counted, mixed 1:1 with ΔCD164 + hCD164^PM^ + eGFP HeLa cells, and seeded into a poly-L-lysine-coated 12-well plate at a density of 2 × 10^5^ cells/well. After overnight incubation at 37°C, the cells were initially imaged using the IncuCyte S3 Live-Cell Analysis System before a 10 min exposure to DMEM-based buffers at pH 7.4, 5.4, or 4.5 with or without 140 mM KCl at 37°C. The pH buffers were then removed, and the cells were neutralized using DMEM supplemented with 2.5% FBS and 20 mM HEPES. After 1 h incubation at 37°C, the cells were imaged using the IncuCyte S3 Live-Cell Analysis System for evidence of cell-cell fusion. Syncytia formation was measured on the IncuCyte S3 Live-Cell Analysis System after an analysis job was performed to identify areas of overlap of green and red fluorescence. Masks of green and red overlap areas were exported from the IncuCyte S3 program and imported into Fiji software. Here, the masks were made binary and inverted, and the number and areas of individual syncytia were measured. The number of syncytia seen and the average area of the syncytia across all images were plotted in GraphPad.

### Statistical analysis

The statistical significance of data was determined by performing Student’s *t*-test. Significance was deemed when the values were less than or equal to the 0.05 *P* value.

## SUPPLEMENTAL MATERIAL

Data Set S1 mbio.01684-23-s0001.xlsx siRNA screen data set to identify ion channels involved in LCMV multiplication.

Supplemental figures mbio.01684-23-s0002.pdf Figures S1-S5.

ASM does not own the copyrights to Supplemental Material that may be linked to, or accessed through, an article. The authors have granted ASM a non-exclusive, world-wide license to publish the Supplemental Material files. Please contact the corresponding author directly for reuse.
