# Supplementary material for: Cellular endosomal potassium ion flux regulates arenavirus uncoating during virus entry
Source: mBio. 2024 Jun 14;15(7):e01684-23. doi: 10.1128/mbio.01684-23 (PMC11253613; doi:10.1128/mbio.01684-23)
Supplement: Supplemental figures — Figures S1-S5. [file mbio.01684-23-s0002.pdf]

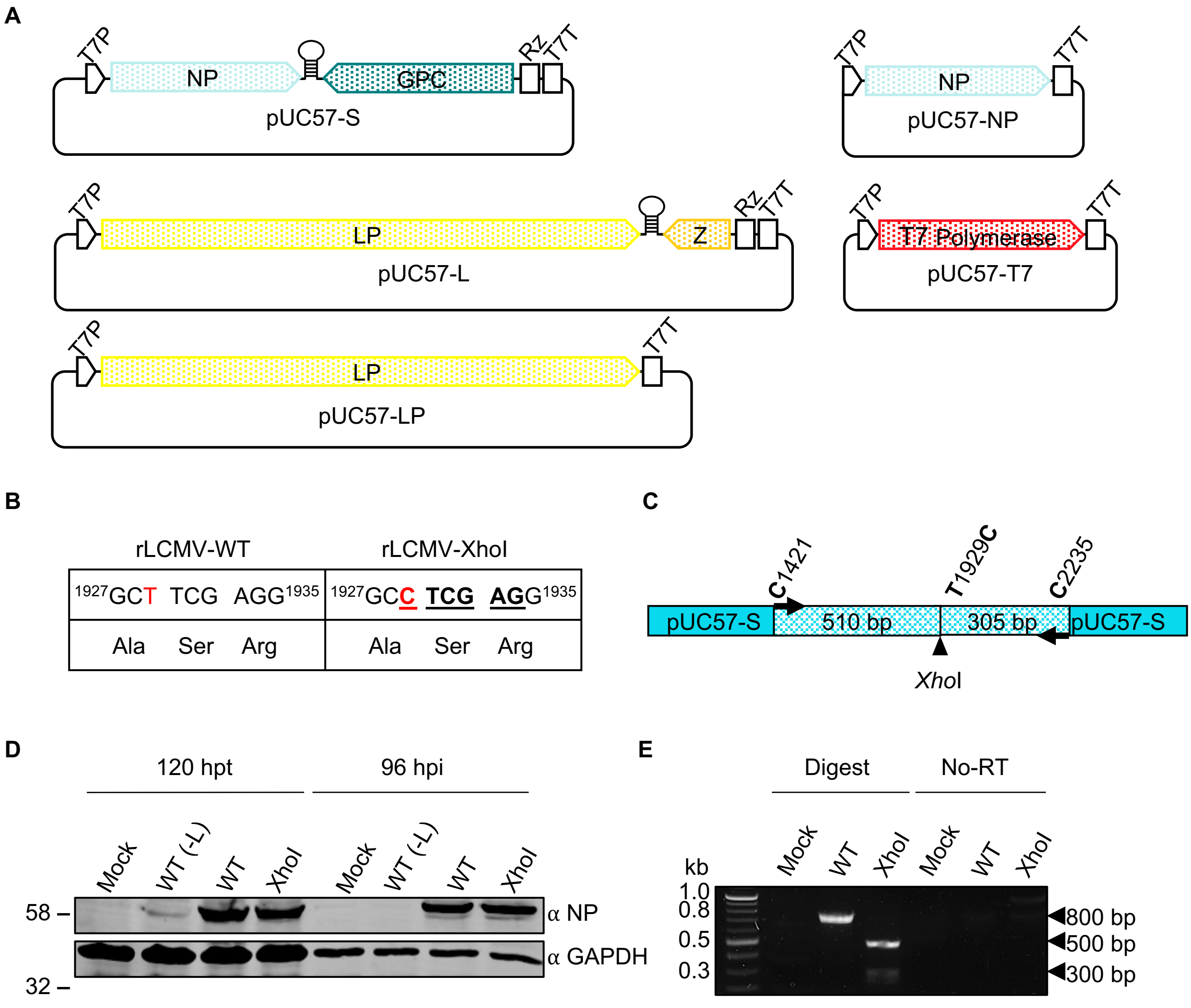

SUPP FIG 1 Development of reverse genetics system to generate recombinant LCMV. (A) Schematic depiction of the plasmids encoding the S segment and L segment of LCMV, in addition to the support plasmids encoding the nucleocapsid protein (NP), the RNA-dependent RNA-polymerase (LP) and the T7 polymerase (T7). Also shown flanking the LCMV S and L segments are the T7 polymerase promoter (T7P), hepatitis delta virus ribozyme (Rz) and the T7 polymerase terminator (T7T) sequences. (B) A silent mutation was introduced to show that the rLCMV-WT derived from the reverse genetics plasmids. T1929 (red) was mutated to C (red) introducing a *XhoI* restriction site (underlined) without disrupting the amino acid sequence. (C) Primers were designed to bind 510 base pairs upstream (C1421) and 305 bp downstream (C2235) from the T1929C mutation in the pUC57-S plasmid. (D) Lysates were collected from BSR-T7 cells transfected with pUC57-L, pUC57-LP, pUC57-NP,pUC57-T7 and either pUC57-S (WT) or pUC57-S-XhoI (XhoI) at 120 hours post transfection and from BHK-21 cells infected with the supernatant from the BSR-T7 cells, 96 hours post infection. Appropriate controls were included whereby the L-expressing plasmids were omitted to prevent rescue of infectious virus (-L). The lysates were probed for NP expression, and GAPDH as a loading control. (E) Supernatant from the infected BHK-21 cells was used to infect fresh BHK-21 cells and at 96 hpi, the supernatant was harvested and RNA was extracted. The extracted RNA was reverse transcribed into complementary DNA and digested with *XhoI* before agarose gel electrophoresis analysis. A control experiment lacking the reverse transcription step was performed, demonstrating the bands were not derived from any contaminating DNA plasmids carried over from the transfected cells.

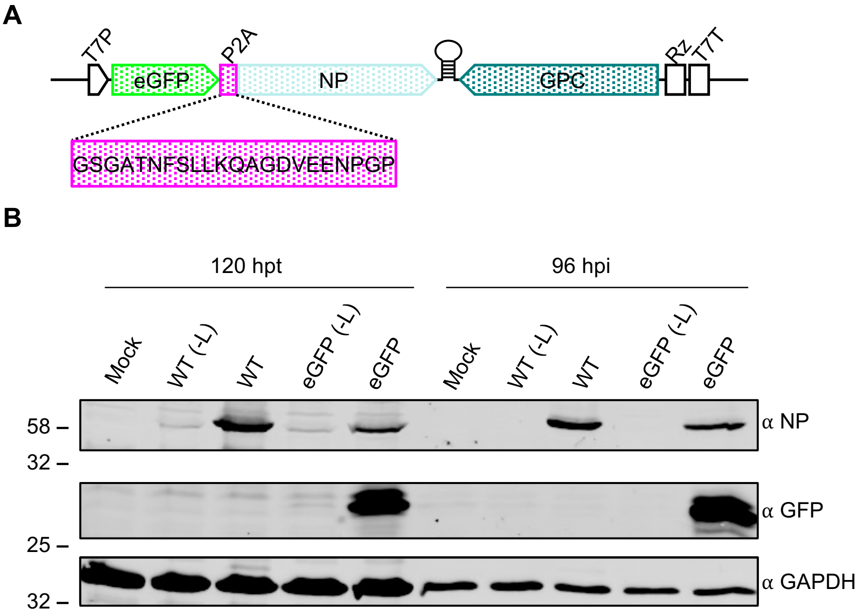

SUPP FIG 2 Generation of an eGFP-expressing variant of LCMV. (A) Schematic depiction of plasmid pUC57-LCMV-S encoding the S segment of LCMV, with the insertion of the eGFP open reading frame upstream of the NP gene. The eGFP and NP genes are separated by a porcine teschovirus 2A linker (P2A), the amino acids of which has been shown in the pink box. Also depicted flanking the LCMV S segment are the T7 polymerase promoter (T7P), hepatitis delta virus ribozyme (Rz) and the T7 polymerase terminator (T7T). (B) Lysates were collected from BSR-T7 cells transfected with the LCMV rescue plasmids 120 hours post transfection (hpt), and from BHK-21 cells infected with the supernatant from the BSR-T7 cells at 96 hours post infection (hpi). Appropriate controls were included whereby the L-expressing plasmids were omitted to prevent rescue of infectious virus (-L). The lysates were probed for NP and EGFP expression, and GAPDH as a loading control using specific antisera, as indicated.

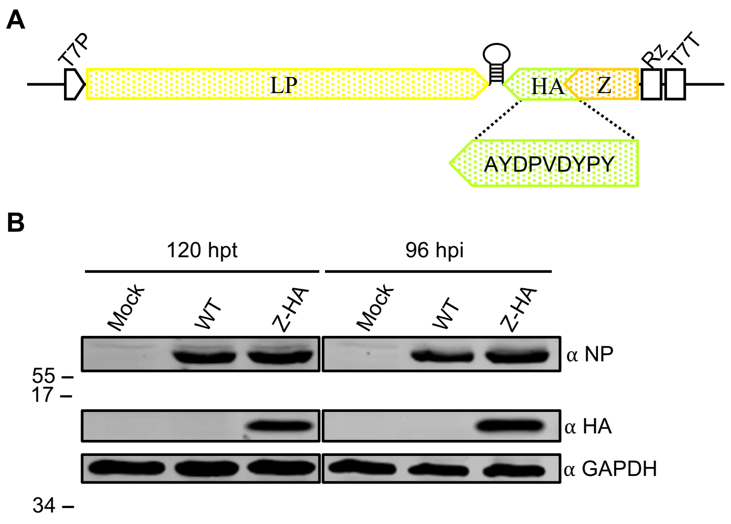

SUPP FIG 3 Rescue of a HA-tagged variant of LCMV. (A) Schematic depiction of plasmid pUC57-LCMV-L-Z-HA encoding the L segment of LCMV, with the insertion of the HA tag (the amino acids of which has been shown in the green box) downstream of the Z open reading frame. Also depicted flanking the LCMV L segment are the T7 polymerase promoter (T7P), hepatitis delta virus ribozyme (Rz) and the T7 polymerase terminator (T7T). (B) Lysates were collected from BSR-T7 cells transfected with the reverse genetics plasmids 120 hours post transfection (hpt) and from BHK-21 cells infected with the supernatant from the BSR-T7 cells, 96 hours post infection (hpi). rLCMV-WT was recovered alongside rLCMV-Z-HA. The lysates were probed for NP and HA expression, and GAPDH as a loading control.

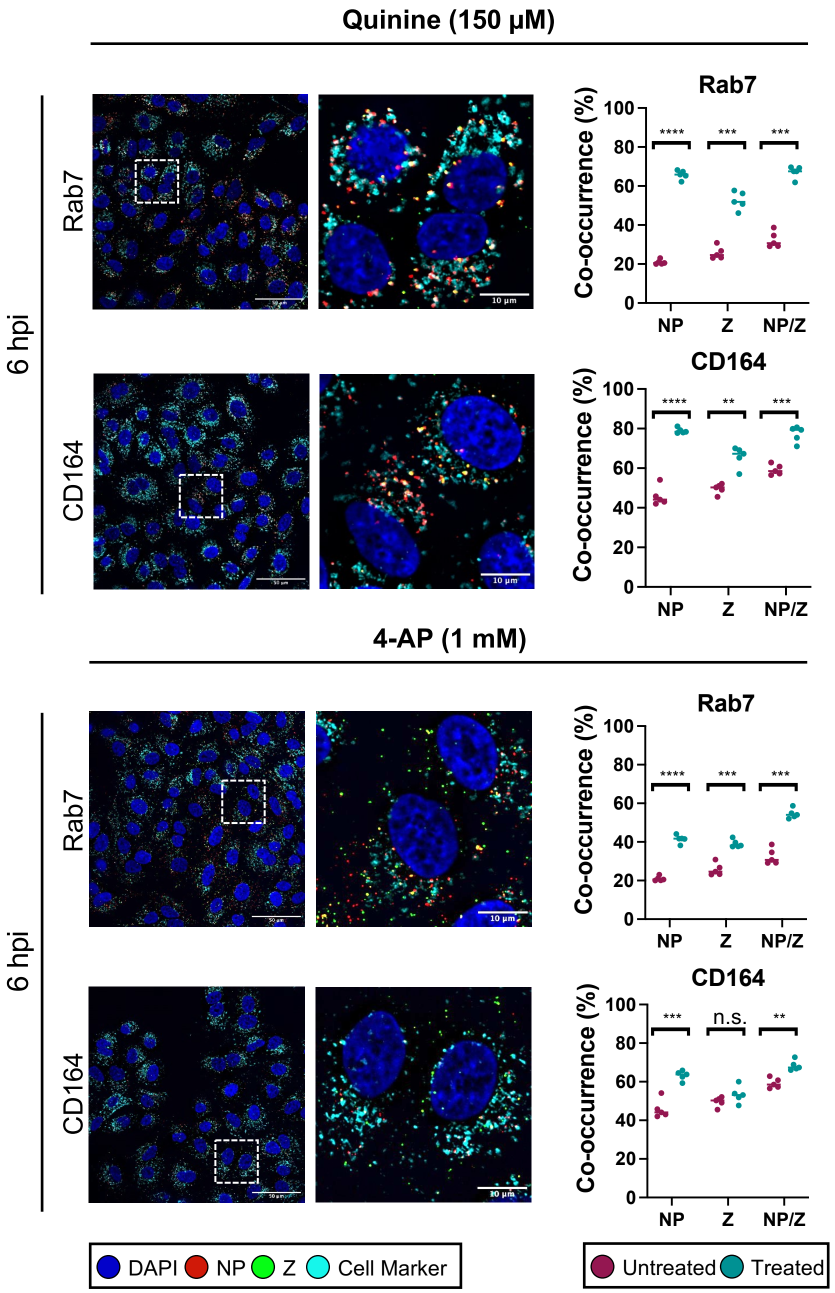

SUPP FIG 4 Treating with broad-acting potassium ion channel inhibitors quinine and 4-AP also cause LCMV NP/Z puncta to co-occur with Rab7. A549 cells were untreated or pretreated with 150 µM quinine or 1 mM 4-aminopyridine (4-AP) and then infected with rLCMV-Z-HA at an MOI of 5 and fixed with formaldehyde at 6 hours post infection (hpi). The cells were then permeabilised, blocked and stained for the nucleus (DAPI; blue), LCMV NP (red), Z-HA (green) and for cellular marker Rab7 (cyan) by indirect immunostaining. The cells were then imaged on the Olympus IX83 widefield microscope at 60 x magnification. White dashed boxes have been included to indicate which region of cells has been shown in the zoomed image. Scale bars representing 50 µm and 10 µm have been included. Alongside the images is co-occurrence analysis, performed over five different images including >150 cells, which has been determined using the Manders' coefficient method. The percentage of either NP signal, Z signal or NP and Z signal has been examined against the signal of the marker and comparison between untreated (pink) and quinidine-treated (blue) was further analysed through the Student's t-test whereby n.s. = p>0.05, * = p<0.05; ** = p<0.01; *** = p<0.001.

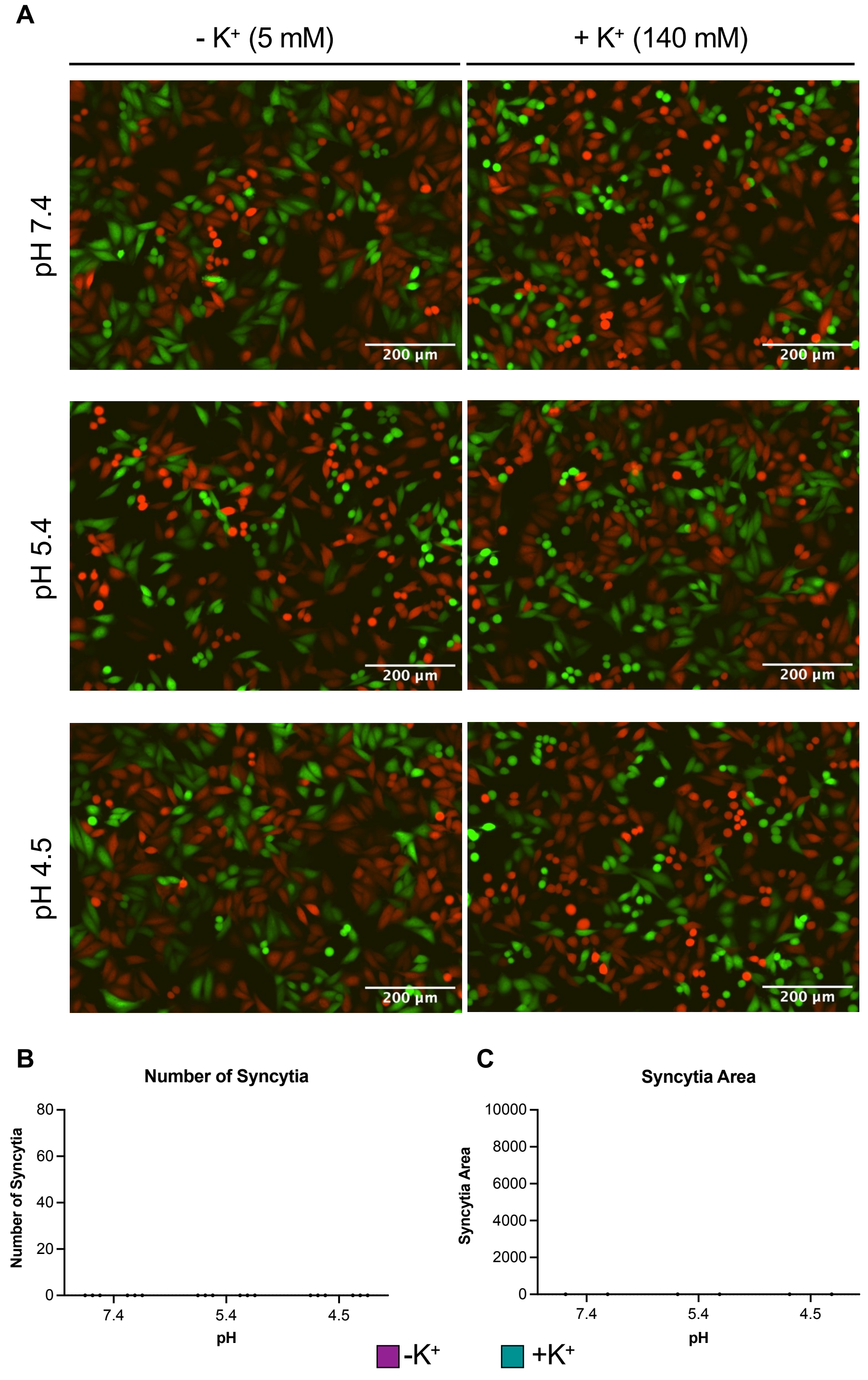

SUPP FIG 5 Fusion does not occur in mock-transfected HeLa cells. (A) ΔCD164 HeLa cells expressing mCherry (red) were mock-transfected (omission of LCMV GPC-expressing plasmid) and at 4 h post-transfection, the cells were washed, trypsinised and mixed 1:1 with ΔCD164 HeLa cells expressing plasma membrane-localised CD164 mutants and eGFP (green) to be seeded onto poly-L-lysine-coated 12 well plates. After overnight incubation, wells were initially imaged. Cells were then washed and treated with media at pH 7.4, 5.4 or 4.5 supplemented with 5 mM potassium chloride (-K) or 140 mM potassium chloride (+K) for 10 mins. The treatment was then removed, and the cells were incubated in media supplemented with HEPES for 1 h. The cells were imaged using the Incucyte S3. From three independent experimental repeats, representative images of green and red fluorescence have been shown. (B) Masks of any green and red overlap areas were exported from the IncuCyte S3 programme and imported into Fiji software. Here, the masks were made binary and inverted and the number and areas of individual syncytia were measured. The number of syncytia seen across all images has been plotted in (B) whereas the average area of the syncytia has been plotted in (C) alongside individual points representing area of each syncytia. All three experimental repeats have been shown.
